# Supplementary material for: Tumor-derived GDF-15 blocks LFA-1 dependent T cell recruitment and suppresses responses to anti-PD-1 treatment
Source: Nat Commun. 2023 Jul 20;14:4253. doi: 10.1038/s41467-023-39817-3 (PMC10359308; doi:10.1038/s41467-023-39817-3)

TUMOR-DERIVED GDF-15 BLOCKS LFA-1 DEPENDENT T CELL RECRUITMENT AND SUPPRESSES RESPONSES TO ANTI-PD-1 TREATMENT

SUPPLEMENTARY INFORMATION FILE

Authors

Markus Haake, Beatrice Haack, Tina Schäfer, Patrick N. Harter, Greta Mattavelli, Patrick Eiring, Neha Vashist, Florian Wedekink, Sabrina Genssler, Birgitt Fischer, Julia Dahlhoff, Fatemeh Mokhtari, Anastasia Kuzkina, Marij J.P. Welters, Tamara M. Benz, Lena Sorger, Vincent Thiemann, Giovanni Almanzar, Martina Selle, Klara Thein, Jacob Späth, Maria Cecilia Gonzalez, Carmen Reitinger, Andrea Ipsen-Escobedo, Kilian Wistuba-Hamprecht, Kristin Eichler, Katharina Filipski, Pia S. Zeiner, Rudi Beschorner, Renske Goedemans, Falk Hagen Gogolla, Hubert Hackl, Rogier W. Rooswinkel, Alexander Thiem, Paula Romer Roche, Hemant Joshi, Dirk Pühringer, Achim Wöckel, Joachim E. Diessner, Manfred Rüdiger, Eugen Leo, Phil F. Cheng, Mitchell P. Levesque, Matthias Goebeler, Markus Sauer, Falk Nimmerjahn, Christine Schuberth-Wagner, Stefanie von Felten, Michel Mittelbronn, Matthias Mehling, Andreas Beilhack, Sjoerd H. van der Burg, Angela Riedel, Benjamin Weide, Reinhard Dummer, Jörg Wischhusen

Correspondence: Wischhusen_J@ukw.de

Supplementary figures:

Figure S1

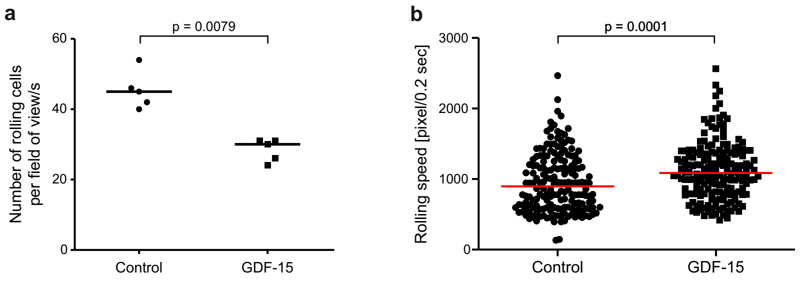

Figure S1: Effect of GDF-15 on T cell rolling and velocity of T cells on HUVEC. a. HUVEC (human umbilical vein endothelial cells) were seeded onto fibronectin-coated µ-slides VI 0.4 (ibidi GmbH, Germany) and left to adhere overnight before being activated with TNF-α and IFN-γ (10 ng/ml each). 24 h later untouched T cells were isolated from human blood. T cells and/or HUVEC cells were then pre-treated with 100 ng/ml GDF-15 for 1h. Under a stage top incubator the T cells were then applied to the channels of the µ-slides and exposed to hydrodynamic flow conditions (0.5 dyn/cm2: 0.38 mL/min = 22.8 mL/h) for 3 min. Each of 10 predefined fields of view was then video-imaged for 5 s and the number of rolling T cells per field of view per second was analyzed. (**b**) Based on the trajectories, the velocity of T cells rolling over HUVEC was determined. Groups were compared using Mann-Whitney test. A representative experiment is shown. Source data for S1a are provided in the source data file.

Figure S2

pan CD4$^+$ T cells — CD8, CD14, CD15, CD16, CD19, CD36, CD56, CD123, TCRγ/δ, and CD235a (glycophorin A)
naïve CD4$^+$ T cells — CD45RO, CD8, CD14, CD15, CD16, CD19, CD25, CD34, CD36, CD56, CD123, TCRγ/δ, anti-HLA-DR, and CD235a
CD4$^+$ memory T cells — CD45RA, CD8, CD14, CD16, CD19, CD56, CD36, CD123, TCRγ/δ, CD235a
pan CD8$^+$ T cells — CD45RA, CD8, CD14, CD15, CD16, CD19, CD34, CD36, CD56, CD123, TCRγ/δd, CD235a, CD197 (CCR7)
CD4$^+$ effector memory T cells — CD4, CD15, CD16, CD19, CD34, CD36, CD56, CD123, TCRγ/δ, CD235a
naïve CD8$^+$ T cells — CD45RO, CD56, CD57, CD244 followed by positive selection via CD8
CD8$^+$ memory T cells — CD4, CD11c, CD14, CD15, CD16, CD19, CD34, CD36, CD45RA, CD56, CD57, CD61, CD123, CD141, TCRγ/δ, CD235a
CD8$^+$ effector memory T cells — CD4, CD11c, CD14, CD15, CD16, CD19, CD34, CD36, CD45RA, CD56, CD57, CD61, CD123, CD141, TCRγ/δ, CCR7^{-depl}
CD4$^+$ regulatory T cells — CD8, CD14, CD15, CD16, CD19, CD36, CD56, CD123, TCRγ/δ, CD235a, CD127^{high} + positive selection for CD25

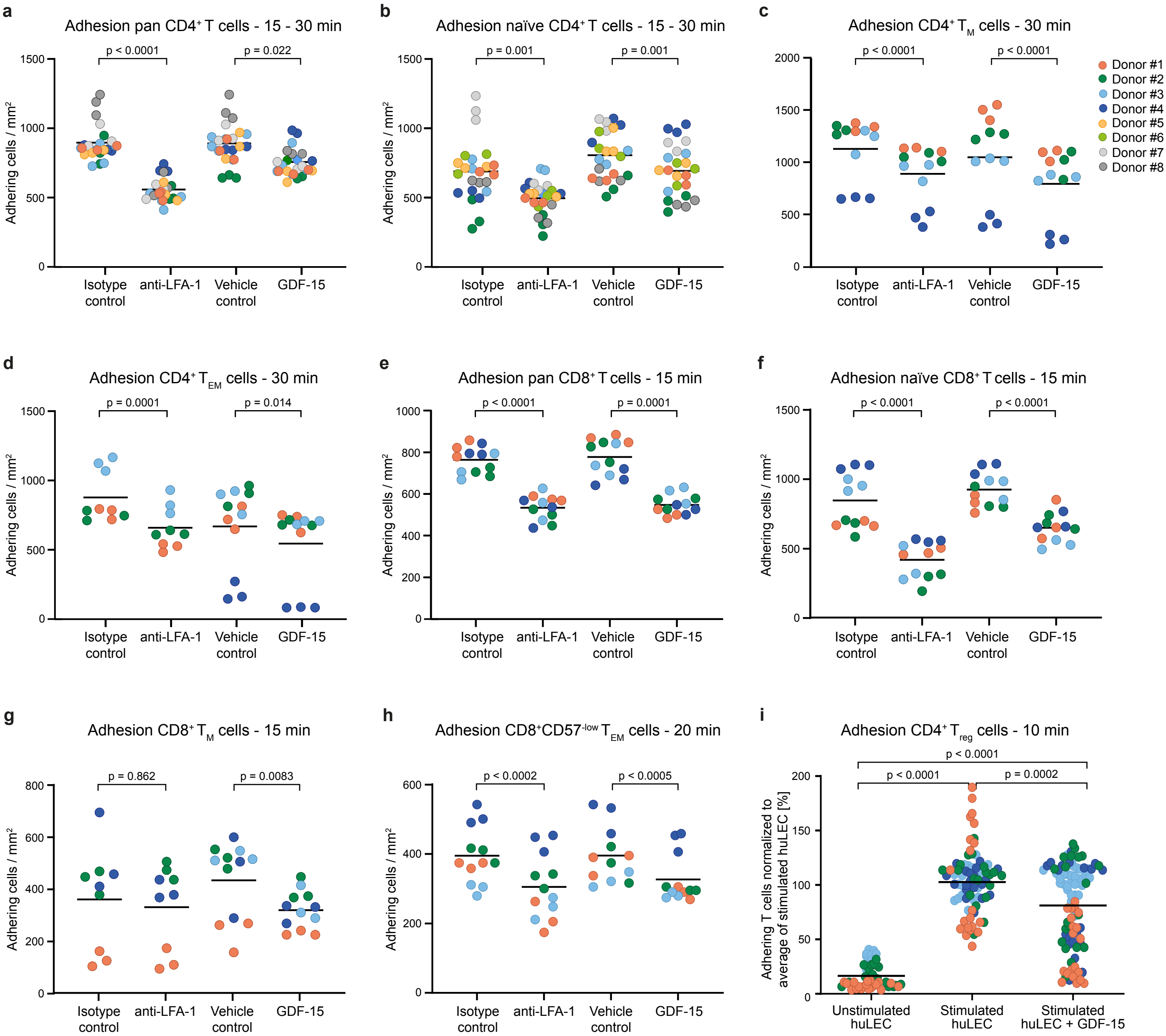

Figure S2. GDF-15 broadly inhibits interaction between T cell subsets and activated endothelial cells.

Negatively selected pan CD4$^+$ T cells **(a)**, naïve CD4$^+$ T cells **(b)**, CD4$^+$ T_M **(c)**, CD4$^+$ T_{EM} **(d)**, pan CD8$^+$ T cells **(e)**, naïve CD8$^+$ T cells **(f)**, CD8$^+$ T_M **(g)** and CD57$^+$ de-enriched CD8$^+$ T_{EM} **(h)** were isolated using magnetic beads and treated or not for 20 min with GDF-15 (100 ng/ml) before being run over activated HUVEC. The assay was set up as in **Figure 1g-i**. The blocking anti-human LFA-1 antibody TS1/18 was used at 20 µg/ml and an isotype was included as positive or, respectively, negative control. (**i**) Activated

and expanded Foxp3$^+$ CD4$^+$CD25$^+$CD127^{dim} regulatory T cells were treated or not with rhGDF-15 for 20 min before being run in μ-slides over a layer of activated huLEC. 10 predefined fields of view were video-imaged for 5s and the number of T cells adhering under hydrodynamic flow conditions was counted. Data points from different experiments with different $T_{effector}$ or T_{reg} donors (4-8 per cell type) are depicted differently shaded. The purity of immune cell subsets was assessed by flow cytometry as per the supplier instructions and ranged between 85-100% for the different immune cell subsets isolated. p values were calculated by one-way ANOVA, and adjusted for multiple comparisons by Tukey´s *post hoc* test. Horizontal lines indicate mean values. Source data for S2a-I are provided in the Source data file.

Figure S3

a GDF-15 expression in human tumor entities

Abbreviation	Study name (cancer type)	No. Normal (TN pairs)	No. Tumor
ACC	Adrenocortical carcinoma	0	79
BLCA	Bladder Urothelial Carcinoma	19	408
LGG	Brain Lower Grade Glioma	0	516
BRCA	Breast invasive carcinoma	109	1093
CESC	Cervical squamous cell carcinoma andendocervical adenocarcinoma	3	304
CHOL	Cholangiocarcinoma	9	36
COAD	Colon adenocarcinoma	26	285
COADREAD (CRC)	COAD+READ	32	379
ESCA	Esophageal carcinoma	11	184
GBM	Glioblastoma multiforme	0	153
GBMLGG	GBM+LGG	0	669
HNSC	Head and Neck squamous cell carcinoma	43	520
KICH	Kidney Chromophobe	25	66
KIPAN	KICH+KIRC+KIRP	129	889
KIRC	Kidney renal clear cell carcinoma	72	533
KIRP	Kidney renal papillary cell carcinoma	32	290
LIHC	Liver hepatocellular carcinoma	50	371
LUAD	Lung adenocarcinoma	58	515
LUSC	Lung squamous cell carcinoma	51	501
DLBC	Diffuse Large B-cell Lymphoma	0	48
MESO	Mesothelioma	0	87
OV	Ovarian serous cystadenocarcinoma	0	303
PAAD	Pancreatic adenocarcinoma	4	178
PCPG	Pheochromocytoma and Paraganglioma	3	179
PRAD	Prostate adenocarcinoma	52	497
READ	Rectum adenocarcinoma	6	94
SARC	Sarcoma	2	259
SKCM	Skin Cutaneous Melanoma	1	103
STAD	Stomach adenocarcinoma	32	415
TGCT	Testicular Germ Cell Tumors	0	150
THYM	Thymoma	2	120
THCA	Thyroid carcinoma	59	501
UCS	Uterine Carcinosarcoma	0	57
UCEC	Uterine Corpus Endometrial Carcinoma	7	176
UVM	Uveal Melanoma	0	80

b

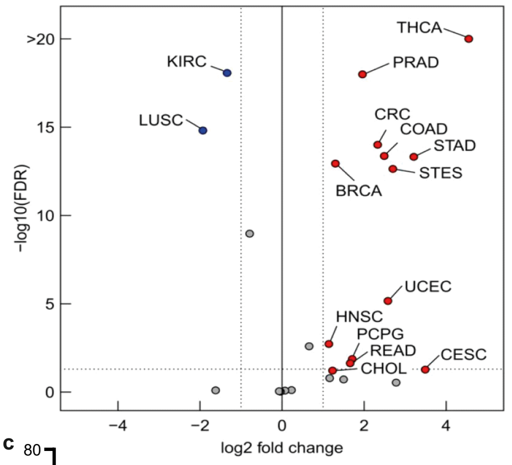

c

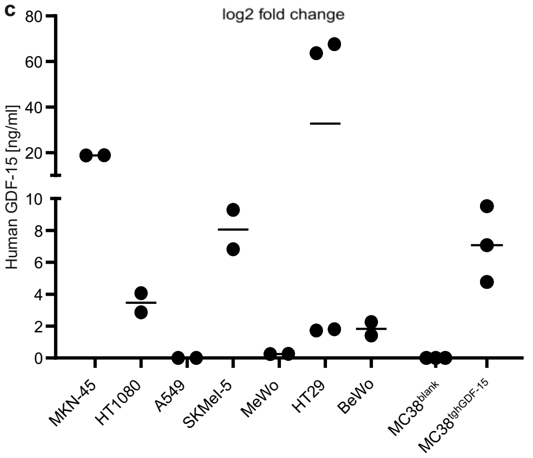

Figure S3: GDF-15 mRNA expression in different human tumor types and GDF-15 protein expression in human tumor cells lines. Gene expression profiles (RNA sequencing V2 data) and clinical information for 33 different cancer type cohorts with additional 3 combined projects of The Cancer Genome Atlas (TCGA) were retrieved via firebrowse.org (courtesy Broad Institute of MIT & Harvard). Samples were filtered for primary solid tumors (sample code 01) and corresponding normal tissue (sample code 11). Using raw count data, differentially expressed genes were identified with the statistical software environment R (version 3.3.2; www.r-project.org) and DESeq2 package. **a**. Respective numbers of analyzed samples from 33 different solid tumor types and 3 combined projects of The Cancer Genome Atlas (TCGA) are presented (top panel). Processed expression data and clinical information were downloaded via firebrowse.org. **b**. Differential GDF-15 mRNA expression between tumor and available corresponding normal tissue using a paired DESeq2 analysis were visualized for different tumor types as volcano plot. **c.** Using the hGDF-15 DuoSet ELISA kit (R&D Systems), concentrations of human GDF-15 were measured in cell culture supernatants from untreated human

tumor cell lines, as well as from murine MC38blank and MC38hGDF-15 cell lines. Cell culture supernatants were collected after 17-24 h (MKN45, HT1080, A549, SKMel-5, MeWo, BeWo) or after 48 h (HT-29, MC38wt, MC38hGDF-15). Horizontal lines indicate median values. Source data for Figure S3a are provided in the Source Data file.

Figure S4

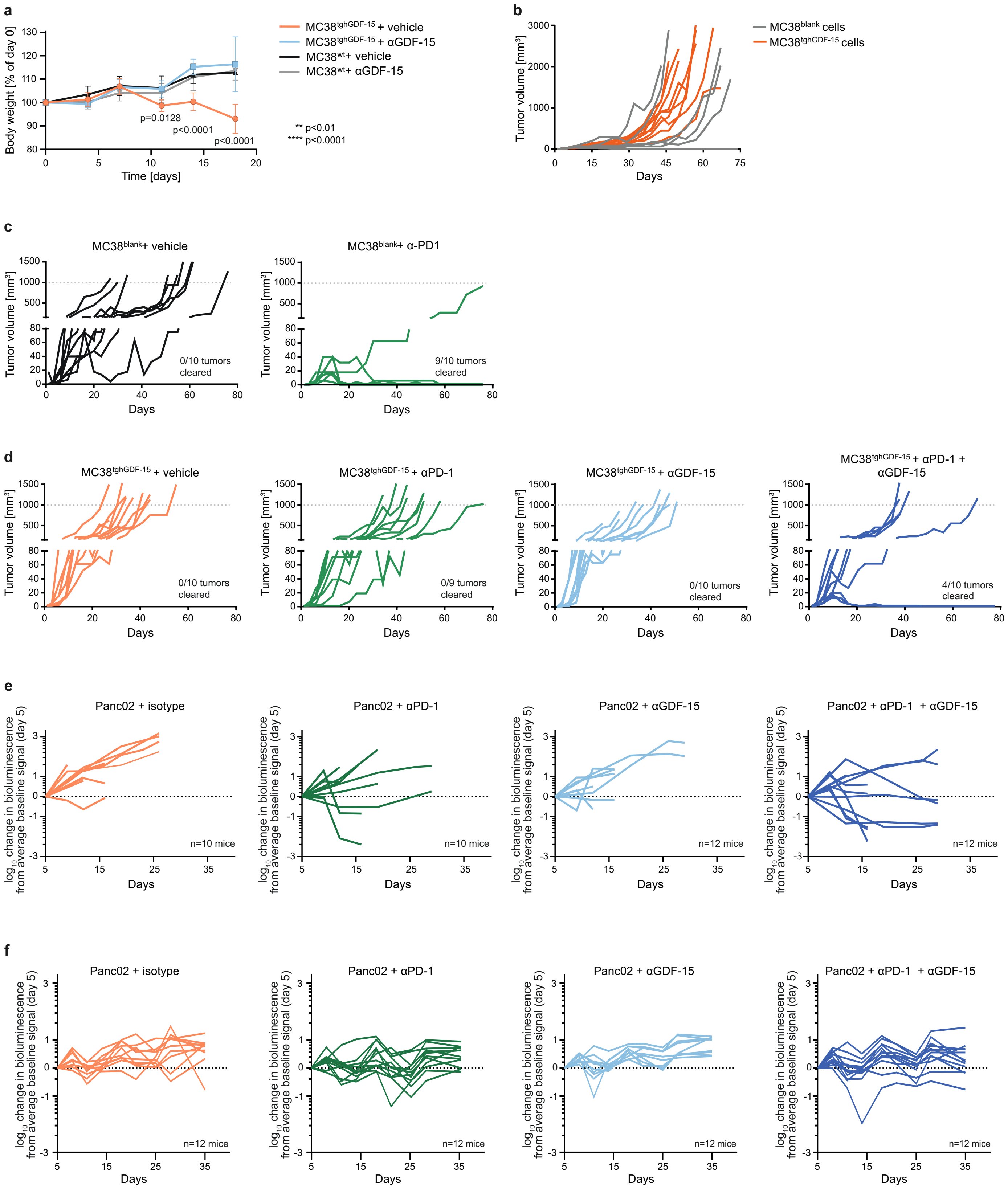

Figure S4: Tumor growth curves from MC38$^{blank/tghGDF-15}$ and Panc02 models. a. To investigate the impact of antibody-mediated GDF-15 blockade on tumor-related cachexia induced by GDF-15, female BALB/c$^{nu/nu}$-mice were subcutaneously inoculated with either MC38$^{wild-type}$ or MC38$^{tghGDF--15}$ cells

(n=20 mice for each cell line). 10 mice each were then treated with anti-human GDF-15 antibody, another 10 mice were injected with vehicle control (PBS with 0.046% BSA). For anti-GDF-15 treatment, 0.5 mg of monoclonal antibody were administered twice weekly via the intraperitoneal route, to achieve a final concentration of 25 mg/kg. Body weight was monitored twice weekly, starting from day 0. Mean values ± SEM are shown. Tumor size was measured on day 7, 11, 14 and 18 using a digital caliper and tumor volume was calculated according to the formula: width^2 x length x 0.5. The experiment was terminated on day 18, when a majority of tumors had reached the maximum size of 1500 mm^3. **b**. 5x10^5 MC38^{blank} or MC38$^{tghGDF\text{-}15}$ cells were injected subcutaneously into the right flank of C57Bl/6NCrl-mice (n=10 mice per group). Tumor sizes were measured by caliper twice weekly. A longitudinal analysis of the tumor volumes estimated a significant difference in the increase in tumor volume over time between the two mouse groups (p=0.0003 for the interaction between group and time, see table S1). **c-f**. Individual tumor growth curves from the experiments shown in Figures **3s,t**, **4a and 4b**. **c,d**. C57BL/6NCrl mice were subcutaneously inoculated with 5×10^5 MC38^{blank} (**c**) or MC38$^{tghGDF\text{-}15}$ (**d**) cells (10 mice per group). Mice were randomized across the different treatment groups and treated or not with anti-PD-1 antibody, in the absence or presence of anti-GDF-15 antibody. In **d**, one mouse from the anti-PD-1 treated group had to be removed and was therefore omitted from analysis. **e,f.** **(e)** 1x10^4 luciferase-transgenic Panc02 cells were inoculated into the pancreatic head of male albino C57Bl/6J mice (10-12 mice/group). In (**f**), 48 female albino C57Bl/6J mice received 1x10^6 Panc02-Luc cells into the pancreatic tail. Animals were randomized into 4 groups (12 mice each) by bioluminescence imaging on day 5, and treated twice-weekly with vehicle/anti-hGDF-15/anti-PD-1/anti-hGDF-15+anti-PD1. Tumor load was assessed at least weekly via bioluminescent *in vivo* imaging. Tumor growth was assessed by relating the last available measurement to the signal during randomization. In (**e**), animals were euthanized on day 29 at the latest, in (**f**) on day 35. Source data for Figure S4a-f are provided in the Source Data file.

Figure S5

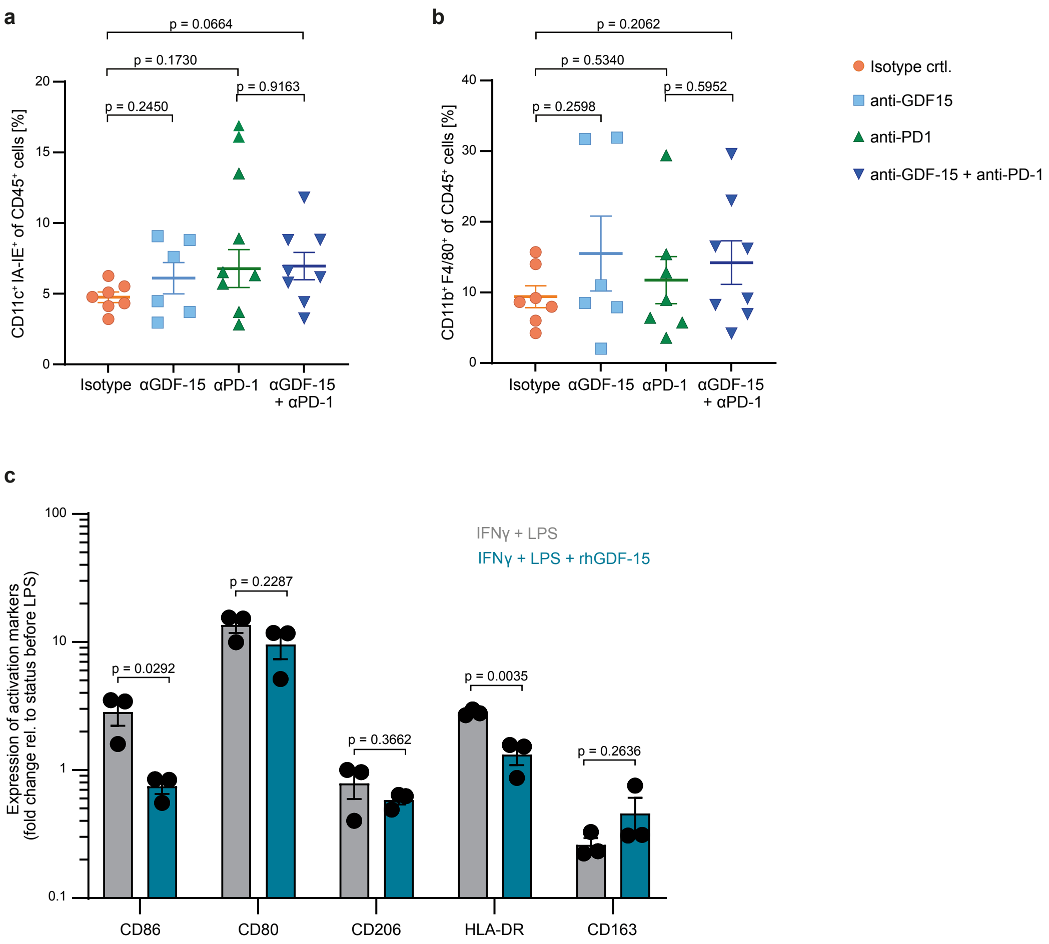

Figure S5. GDF-15 and anti-PD-1 blockade do not affect the presence of macrophages and dendritic cells in established orthotopic Panc02 tumors. 1x10^4 luciferase-transgenic Panc02 cells were inoculated into the pancreatic head of male albino C57Bl/6J mice. After bioluminescence-based randomization on day 5, 32 animals were treated twice-weekly with vehicle/anti-hGDF-15/anti-PD-1/anti-hGDF-15+anti-PD1. 4 animals had to be euthanized due to non-tumor related reasons. On day 12, the remaining 28 animals were euthanized. Tumors were explanted and dissociated to analyze the presence of CD11c$^+$ IA-IE (MHC class II)$^+$ dendritic cells (**a**) and of CD11b$^+$ F4/80$^+$ macrophages (**b**) by flow cytometry. (**c**) Characterization of M-CSF-induced, monocyte-derived macrophages that were matured with IFN-γ, LPS and (where indicated) rhGDF-15. Expression of maturation markers was assessed by flow cytometry (n=3 donors). Mean ± SEM are shown. P values were computed by two-sided, unpaired Student´s t test. Source data for Figure S5a-c are provided in the Source Data file. Flow cytometric gating strategies applied for Figures S5a,b are provided as Supplementary information.

Figure S6

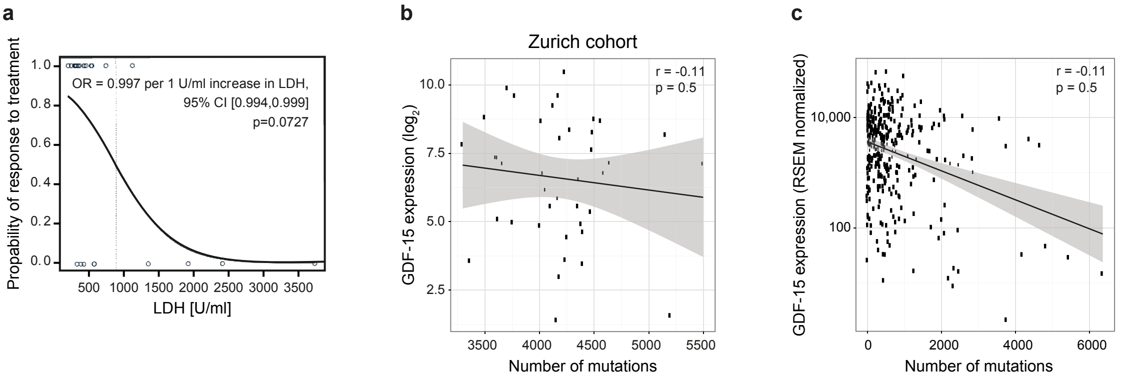

d

Cohort	Identifier	platform	n	Correlation GDF-15 – PD-L1	
				R	p
Cutaneous melanoma (primary & metastatic)	ps_avgpres_tcgaskcm470_tcgars	tcgars	470	-0.046	0.319
	Cancer Genome Atlas (2015). Genomic Classification of Cutaneous Melanoma. Cell 161(7), 1681-1696.	tcagrs	375	-0.074	0.151
	ps_avgpres_tcgaskcmv32a473_gencode36	gencode36	473	-0.107	**0.020**
Metastatic melanoma prior to anti-PD-1	Hugo, W et al. (2016). Genomic and Transcriptomic Features of Response to Anti-PD-1 Therapy in Metastatic Melanoma. Cell 165(1), 35-44.	RNAseq	28	-0.255	0.190

Figure S6: Prediction of response to anti-PD-1 treatment by serum lactate dehydrogenase (LDH) levels, and lack of correlation between GDF-15 expression and tumor mutational burden. a. The response to anti-PD-1 treatment (binary variable) of 29 patients was analyzed by logistic regression, with LDH (U/ml) as continuous predictor. Response to treatment was assessed by RECIST1.1 criteria after 4 months. The model estimated treatment an OR of 0.997 (95% CI [0.994,0.999], per U/ml LDH, p=0.0727) 4 blood samples had to be excluded where the determination of LDH failed due to hemolysis. **b.** Based on TruSeq RNA v2 (RS-122-2001, Illumina) paired end sequencing, GDF-15 expression and the number of mutations were determined for 40 melanoma patients from Zurich (**b**) and for a TCGA-based set of 344 melanoma patients (**c**). The number of coding mutations was plotted on the x- and the normalized GDF-15 expression on the y-axis. Gene expression profiles from the Zurich cohort have been deposited under https://www.ncbi.nlm.nih.gov/geo/query/acc.cgi?acc=GSE198776. The analyzed TCGA data had been retrieved from https://portal.gdc.cancer.gov/. The error bands

shown in **b** and **c** represent the 95% confidence interval for the correlation. (**d**) Using the "R2: Genomics Analysis and Visualization Platform (http://r2.amc.nl)", an *in silico* correlation analysis was performed to test whether GDF-15 mRNA expression correlates with PD-L1/CD274 mRNA expression in melanoma. Correlation coefficients and p values from 4 different clinical cohorts are shown. Source data for Figure S6a are provided in the Source Data file.

Figure S7

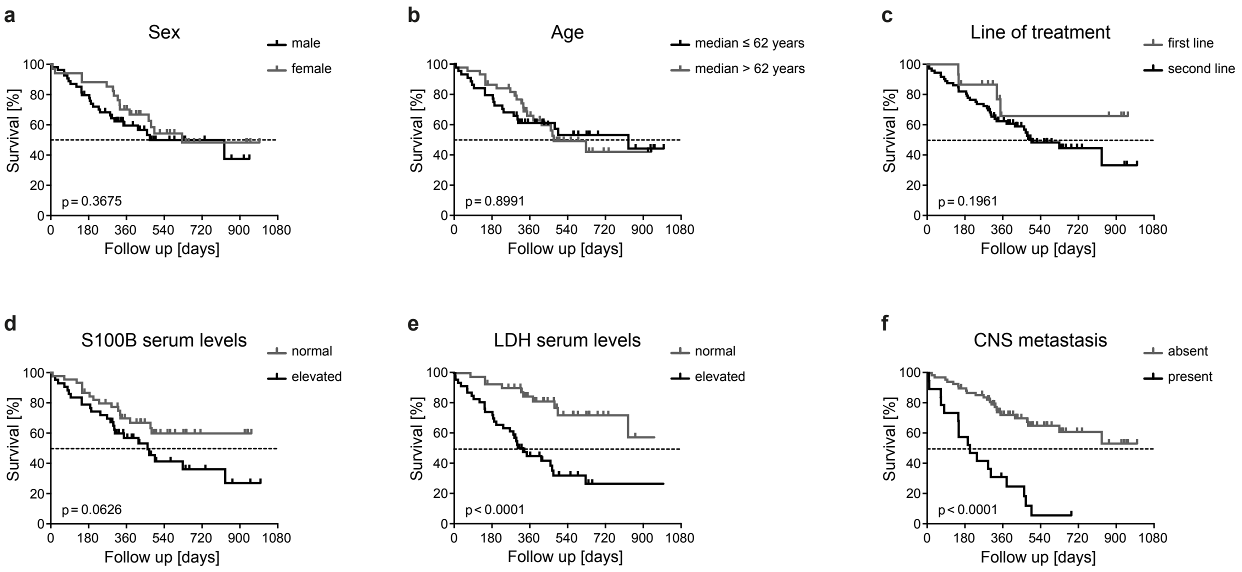

g

	Categories	n	HR	95% CI	p
**Age**	=62 years	44			0.931
	>62 years	44	0.967	0.450-2.079	
**Sex**	female	34			0.091
	male	54	1.797	0.910-3.547	
**Lineof treatment**	first	15			0.673
	second	73	1.267	0.421-3.814	
**CNS metastasis**	absent	69			**<0.001**
	present	19	6.121	2.977-12.587	
**LDH serum levels**	normal	41			**0.008**
	elevated	47	3.255	1.352-7.840	
**S100B serum levels**	normal	45			0.315
	elevated	43	1.494	0.683-3.268	
**GDF-15 serum level**	<2 ng/ml	60			**0.001**
	≥2 ng/ml	28	3.312	1.617-6.783	

Global p-value (chi-square) <0.001

h

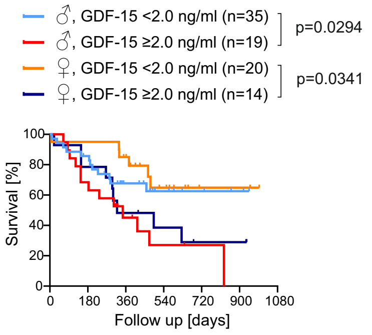

Figure S7: Multi-variate analyses of survival of anti-PD-1 treated melanoma patients from the Tübingen cohort. 88 melanoma patients scheduled for treatment with anti-PD-1 (pembrolizumab: n=48; nivolumab: n=40) were dichotomized according to (**a**) sex (female/male), (**b**) age (below or above median), (**c**) line of treatment (first/second), (**d**) S100B serum levels (normal/elevated), (**e**) LDH serum levels (normal/elevated) and (**f**) absence or presence of brain metastasis. Shown are curves depicting overall survival from onset of treatment. (**g**) In multi-variate analyses, Cox proportional hazard models were used to study the relationship between predictor features and overall survival time. The results are described by means of hazard ratios and p-values (Wald test). Source data for Figure S7a-f are provided in the Source Data file. (**h**) To check for sex-specific effects of GDF-15 in melanoma, separate survival curves were plotted for female and male patients, dichotomized by GDF-15 levels < or ≥ 2.0 ng/ml. p values for GDF-15^{low} vs. GDF-15^{high} in female and male melanoma patients were calculated by Mantel-Cox (log-rank) test.

Figure S8

a

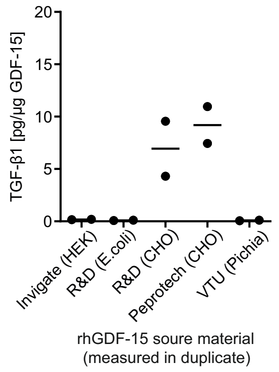

b

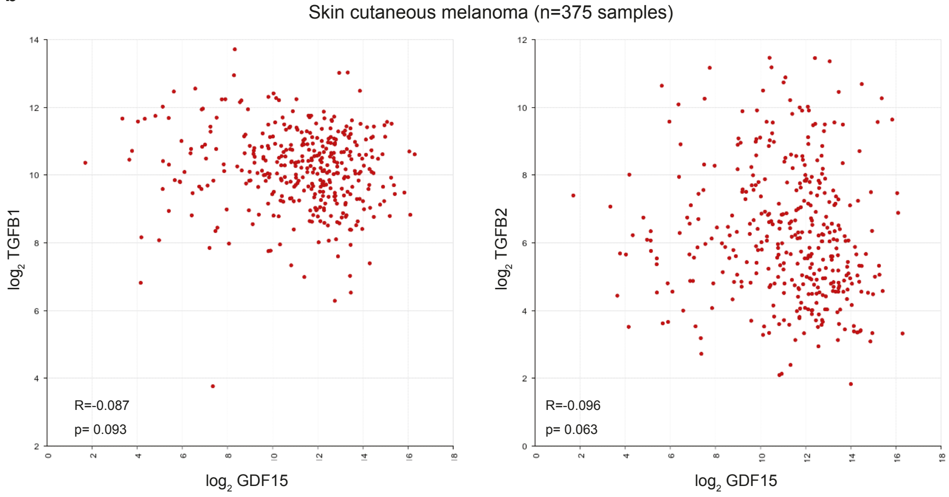

c

Cohort	Identifier	platform	n	Correlation GDF-15 vs.TGFB1		Correlation GDF-15 vs.TGFB2	
				R	p	R	p
Cutaneous melanoma (primary & metastatic)	ps_avgpres_tcgaskcm470_tcgars	tcgars	470	-0.058	0.213	-0.129	**<0.005**
	Cancer Genome Atlas (2015). Genomic Classification of Cutaneous Melanoma. Cell 161(7), 1681-1696.	tcagrs	375	-0.087	0.093	-0.096	0.063
	ps_avgpres_tcgaskcmv32a473_gencode36	gencode36	473	-0.106	**0.021**	-0.084	0.067
Metastatic melanoma prior to anti-PD-1	Hugo, W et al. (2016). Genomic and Transcriptomic Features of Response to Anti-PD-1 Therapy in Metastatic Melanoma. Cell 165(1), 35-44.	RNAseq	28	-0.133	0.499	-0.246	0.207

Figure S8: Analysis of possible relationships between TGF-β and GDF-15 with a potential impact on our study. a. To ascertain that effects observed with rhGDF-15 could not be due to contamination with TGF-β, rhGDF-15 from different manufacturers was analyzed for the presence of TGF-β, using the quantikine human TGF-β1 ELISA kit (R&D Systems, # DB100B). For experiments shown in this manuscript HEK293 cell-derived GDF-15 from Invigate (Jena, Germany) was used. **b,c**. Using the "R2: Genomics Analysis and Visualization Platform (http://r2.amc.nl)", we have performed *in silico* correlation analyses to test whether GDF-15 mRNA expression correlates with TGFB1 or TGFB2 mRNA expression in melanoma. **b**. Representative plots depicting the relationship between gdf15 and tgfb1 or, respectively, tgfb2 are shown. **c**. Correlation coefficients and p values from 4 different clinical cohorts are shown. Source data for Figure S8a are provided in the Source Data file.

Figure S9

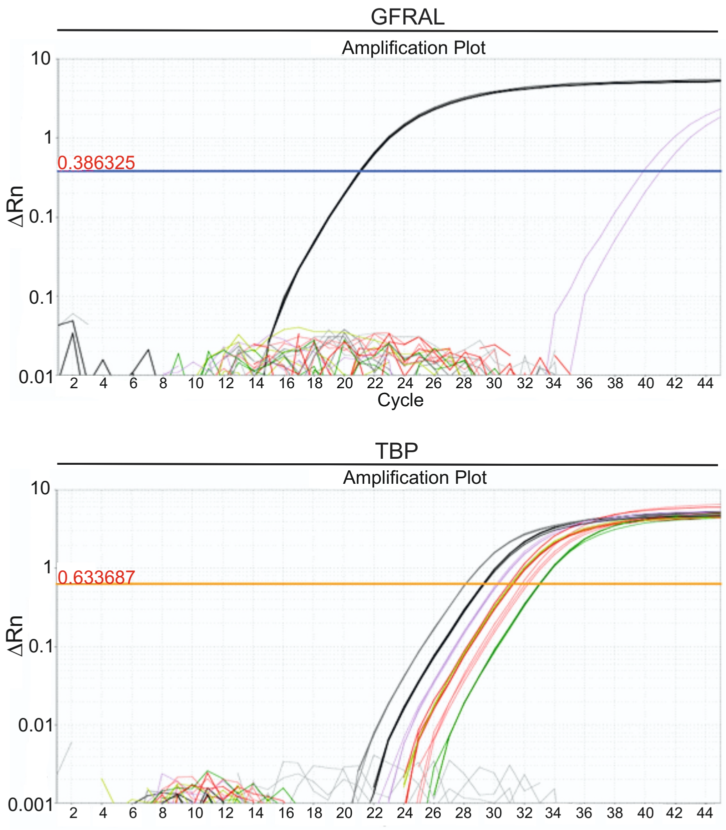

Figure S9: GFRAL can be detected by qRT-PCR in human microvascular endothelial cells (huMEC), but not in human T cells or PBMC. Upper panel. Analysis of GFRAL gene expression in HEK293 (grey) cells and in HEK293 cells transiently transfected with GFRAL (black) as well as in PBMC (light blue), T cells (green) and huMEC (violet). Lower panel. TATA-Box binding protein (TBP) served as housekeeping gene control. x-axis: number of cycles; y-axis: normalized reporter (Rn) value of an experimental reaction minus the Rn value of the of the baseline signal generated by the instrument.

Supplementary Table 1: Estimates from the linear mixed-effects model fitted to the data shown in Figure 3d. Tumor volumes in mm^3 (outcome variable), taken until mice had to be euthanized or up 74 days, were analyzed with mice group (mice with $MC38^{tghGDF\text{-}15}$ cells vs mice with $MC38^{blank}$ cells) and day as fixed explanatory variables and a random intercept per mouse to account for the non-independence of tumor volumes from the same mouse. On day 36 (days were centered to 36), tumor volumes of mice with $MC38^{tghGDF-15}$ cells were on average 668.4 mm^3 larger and increased faster: whereas tumor volumes of mice with $MC38^{blank}$ cells increased by 14 mm^3 per day, those of mice with $MC38^{tghGDF\text{-}15}$ cells increased by 14.2 + 21.6 = 35.7 mm^3 per day. The model included 371 measurements from 20 mice.

Model Term	Estimate	95 % Confidence Interval	t-Value	p-Value
(Intercept)	452.55	from 35.36 to 869.73		
Group GDF-15 vs. Blank	668.44	from 29.18 to 1307.70	2.197	0.041
Day (centered)	14.18	from 6.49 to 21.87	3.628	0.0003
Group GDF-15 vs. Blank x Day	21.57	from 10.09 to 33.05	3.696	0.0003

Supplementary Table 2: Cox proportional hazards regression model for cohort shown in Figure 6.

Table S2 shows the HR estimates from three Cox models on overall survival on the two different clinical cohorts. For the Zurich cohort, one analysis was performed. The Tübingen cohort was first analyzed considering all patients (n=88). In a sensitivity analysis, two patients with GDF-15 > 100 ng/ml were excluded. Overall survival was considered as outcome variable, GDF-15 (in ng/ml) as continuous predictor. While GDF-15 levels ranged from 0.345 ng/ml to 12.010 ng/ml in the Zurich cohort (shown in **Figure 6c-e)**, GDF-15 levels in the Tübingen cohort (shown in **Figure 6f-j**) ranged from undetectable to 1640 ng/ml, or from undetectable to 34.63 ng/ml, if the two patients with the most extreme GDF-15 concentrations are excluded. Consequently, hazard ratios per unit change in GDF-15 (measured in ng/ml) are much smaller in the Tübingen cohort. Still, the hazard of death is significantly increased at higher concentrations of GDF-15 in both cohorts and analyses.

1st cohort, all values (n=34) included					
Predictor	range	HR	95% CI	z	p
ng/ml GDF-15	[0.345-12.010]	1.27	[1.10, 1.47]	3.27	0.00109
2nd cohort, all values (n=88) included					
Predictor	range	HR	95% CI	z	p
ng/ml GDF-15	[0.00-1640]	1.002	[1.00, 1.004]	3.303	<0.001
2nd cohort, excluding 2 patients with GDF-15 > 100 ng/ml (n=86)					
Predictor	range	HR	95% CI	z	p
ng/ml GDF-15	[0.00-34.63]	1.057	[1.02, 1.100]	2.710	0.00672

Supplementary Methods in the form of extended Figure captions

Figure 1. GDF-15 interferes with T cell adhesion to activated endothelial cells. **a.** CXCL12α (200 ng/ml)-mediated adhesion of whole blood-derived $CD45^+$ cells to activated human lymphatic endothelial cells (huLEC) was analyzed in the absence or presence of 10 ng/ml rhGDF-15 (added for 10 min). While non-adherent cells were removed by aspirator-based washing with PBS (with Ca^{2+} and Mg^{2+}) at 37°C, adhering cells were subsequently detached with ice-cold PBS without Ca^{2+} and Mg^{2+}. Adherence was calculated based on the number of $CD45^+$ cells as enumerated by flow cytometric analysis (n=7). **b.** Whole blood was treated with CXCL12α ± GDF-15 as in **a** and cells were allowed to adhere to huLEC. Adhering leukocytes were stained, fixed over-night with 4% para-formaldehyde, and enumerated using an Attune NxT flow cytometer (n=10 experiments). **c.** huLEC were seeded onto fibronectin-coated µ-slides VI 0.4 (ibidi GmbH, Germany) and left to adhere overnight before being stimulated (or not) with TNF-α and IFN-γ (10 ng/ml each for 20-24 h). Untouched T cells were isolated from human blood and activated overnight (20-24 h) with ImmunoCult™ (antiCD3/CD28/CD2) before being treated with 10 ng/ml GDF-15 or vehicle for 20 min. Under a stage top incubator, T cells were applied to the channels of the µ-slides and exposed to hydrodynamic flow conditions (0.5 dyn/cm^2 to obtain a flow rate of 22.8 ml/h) for 3 min. Each of 10 predefined fields of view was then video-imaged for 5s and the number of adhering T cells per field of view per second was analyzed. Data points from different experiments with different T cell donors are depicted in different colors. **d.** Human umbilical vein endothelial cells (HUVEC) were seeded onto fibronectin-coated µ-slides VI 0.4. 24 h later untouched T cells were isolated from human blood. T cells and/or HUVEC were then pre-treated with 100 ng/ml GDF-15 for 1h. Adhesion and rolling of T cells were determined as in **c**. **(e-j).** HUVEC were cultured in chamber slides for 2 days, activated over night with TNF-α and IFN-β. On the day of the experimentation HUVEC were equilibrated for 5 min with chemokines (CXCL12α or CXCL9 + CXCL10) and GDF-15 used at the indicated concentration, followed by a 15 min stasis before being washed. T cells purified via negative bead-based selection were treated for 20 min with GDF-15 (100 ng/ml) or vehicle control, before being perfused for 6 min over the stimulated HUVEC layer. Then, wash buffer

was run at constant pressure over the cells for the next 50 min. Phase contrast microscopy images of leukocytes captured in fixed fields were recorded every 30 s. Leukocytes adhering to the HUVEC layer had a phase-white/grey appearance, whereas those that had transmigrated (recruited) showed a phase-black appearance. Transmigration events (phase black) are presented as a percentage of total T-cell (phase grey + black) captured from flow per unit field **e.** T cell adhesion was stimulated by CXCL9 + CXCL10 (used at 4 µg/ml each) and antagonized by 100 ng/ml GDF-15. **f.** stimulated $CD8^+$ T cells were treated with rhGDF-15 and anti-GFRAL or isotype control antibodies for 20 min before being run in µ-slides over a layer of activated huLEC. **g.** GDF-15 was applied at different concentrations. The respective effect on T cell adhesion in the presence of CXCL12 was quantified for T cells from 3 different donors, and a dose-response curve was generated by integrating the respective mean values ± SEM for adhesion over 50 min. **h,I,j.** Using T cells from 9 different donors, effects of 100 ng/ml GDF-15 on T cell adhesion (**h**), transmigration (**i**) and recruitment (**j**) were analyzed at 40 min. Statistical analyses were performed by one-way ANOVA in **a,d,f**, by two-sided paired Student´s t tests in by **b**,**e,h,i,j**, by mixed-effects analysis in **c**. To correct for multiple comparisons, Tukey´s post-hoc test was applied in **a,c,d**, Bonferroni´s method in **b**. In **g**, an EC_{50} value for rhGDF-15-mediated adhesion inhibition on pan T cells from 3 different donors was determined according to the formula (logICF=logIC50 + (1/HillSlope)*log(F/(100-F)). In **c,d,g**, mean values with SEM, in **e,f**, median values are indicated as horizontal lines. Source data are provided as a source data file. Gating strategies applied for Figures 1a and b are provided as Supplementary information.

Figure 2. GDF-15 interferes with LFA-1-dependent adhesion of human T cells. In **a**, slides were coated (or not) with ICAM-1-Fc and CXCL12α. In **b**, slides were coated with CXCL12α and either MAdCAM-1-Fc or ICAM-1-Fc. In **c**, slides were coated with CXCL12α and either VCAM-1-Fc or ICAM-1-Fc. Activated T cells were either treated or not with rhGDF-15 (100 ng/ml). Blocking antibodies against LFA-1 (TS1/18, used at 20 µg/ml), α4β7 integrin (MAB10078, used at 5 µg/ml), or VLA-4 VLA-4 (BE0071, used at 20 µg) were added as indicated. In **d,e**, $CD4^+$ and $CD8^+$ T cells were pre-treated for 20 min with GDF-15 (100 ng/ml), or blocking anti-LFA-1 antibody TS1/18 (20 µg/ml), or both, and run over activated

HUVEC cells. Technically, adhesion assays with HUVEC were conducted as in (**1e-i**). In **a-e**, the actual numbers of adhering T cells per field of view were counted under flow conditions. In **f,g,** LFA-1 mediated ligand capture was assessed for $CD8^+$ T cells in whole blood from healthy volunteers, by applying either a conformation-specific anti-active LFA-1 antibody mAb24 (**f**) or ICAM-Fc, complexed with an APC-conjugated $F(ab)_2$ anti-human Fc (**g**). Human blood was maintained at 37°C and either treated or not with 20 ng/ml rhGDF-15 GDF-15 for 10 min prior to addition of the stimulation (200 ng/ml CXCL12α, Immunocult αCD3/CD28 used at 1:20 dilution, $Mg2^+$ added to 1 mM or, where indicated, 2 mM) and staining (anti-CD3-APC/Cy7, anti-CD4-BrilliantViolet711, anti-CD8-PECy7). Following 10 min incubation with stimulants, fluorescence-conjugated antibodies and complexed APC-labeled soluble ICAM-Fc, cells were fixed and analyzed on an Attune Nxt flow cytometer. Samples were measured in duplicates. Median values are indicated as horizontal lines. **h,i,j.** Human peripheral blood leukocytes were stimulated with 100 ng/ml CXCL12α and 1 mM Mg^{2+} and 1 mM EGTA for 30 min in the absence or presence of 10 ng/ml rhGDF-15. Cells were stained for 20 min with the conformation-specific Alexa Fluor 647-labeled anti-LFA-1 antibody mAb24 (5 µg/ml) (**h**) or with an immune complex consisting of hICAM-1-Fc (5 µg/ml) together with an Alexa Fluor 647 conjugated anti-human IgG antibody (5 µg/ml) (**i**). A precise quantification of the number of active LFA-1 molecules per single $CD3^+$ T cell was performed by *d*STORM imaging with subsequent quantification of localizations per cluster. Representative *d*STORM images are shown in **h,i**. Data obtained with mAb24 across three different donors are summarized in **j**. **k,l**. 3 mg Protein G beads (Dynabeads™, thermofisher) were loaded with 24 µg recombinant human ICAM-Fc and 24 µg recombinant E-Selectin-Fc and washed. $4x10^6$ purified T cells were added to 1.5 mg ICAM-coated beads in a total volume of 200 µl PBS with Ca^{2+} and Mg^{2+}, in the absence or presence of 100 ng/ml GDF-15. After 7 min, cells were lysed by adding 100 µl hot lysis buffer (1 % SDS,10 mM Tris pH 8.0, 1 mM EDTA, 10 mM PMSF, 25 mM β-Glycerophosphat, 1 mM NaF, 1mM NaV_2O_5, all from Sigma, Proteinase Inhibitor (Carl Roth, Karlsruhe, Germany) and PhosStop (Roche)). After sonification, beads were magnetically removed and protein concentrations were determined by Bradford assay. 20 µg protein/lane were loaded on an SDS-polyacrylamide gradient gel

(7.5-15%), separated by SDS gel electrophoresis, transferred onto a PVDF membrane and probed with anti phospho-Talin (Ser^{425}) (clone D2P2M, Cell Signaling) and anti-CD3ε (Clone UCHTI, Biolegend) and hrp-labeled secondary antibodies (Anti-rabbit IgG, HRP linked Antibody, Cell Signaling #7074S, Anti-mouse IgG, HRP linked Antibody, Cell Signaling #7076S). Signals were detected in an iBright CL750 chemoluminescence imager (thermofisher), using Westar Supernova (Cyanagen, Bologna, Italy). The pTalin band runs at ~250 kD, the CD3-specific signal at ~20 kD. A representative blot is shown in **k**. Protein quantification data from 7 different samples were normalized to the vehicle (HAS) control and are displayed in **l**. Statistics were calculated by Kruskal-Wallis with Dunn´s post hoc test (**a**), by one-way ANOVA with Tukey´s correction for multiple comparisons (**b-e**), and by two-sided paired t tests (**f,g,j,l**). Horizontal bars indicate mean (**a,f,g**) or median (**d,e,j,l**) values. Source data for Figures 2a-g,j,l are provided as source data file. An unprocessed image of the Western Blot shown in Figure 2k and flow cytometric gating strategies applied for Figures 2f,g are provided as Supplementary information.

Figure 3. GDF-15 interferes with immune infiltration and immune-mediated tumor rejection in the MC38 colon cancer model *in vivo*. a,b. $5x10^5$ $MC38^{blank}$ or $MC38^{tghGDF-15}$ colon cancer cells were injected subcutaneously into the right flank of $NCI^{nu/nu}$-mice. The body weight of tumor-bearing mice was determined twice per week **(a)**. Tumor growth was also determined twice weekly by caliper measurement **(b)**. Data are based on 6 mice per group from a representative experiment (n=4 independent experiments). **c-h.** Tumor take rates were recorded from 4 different experiments, based on 10 mice per experiment **(c)**. Tumor growth curves for the two different MC38 sublines were compared in 4 different experiments based on 10 mice per group. A Kaplan-Meier plot for a representative experiment (with a termination criterion of tumor size ≥ 1200 mm^3) is shown in **(d)**. Using 6 mice per group, sera from C57Bl/6NCrl mice inoculated with $MC38^{blank}$ or $MC38^{tghGDF-15}$ tumors were obtained on day 28. Levels of human GDF-15 were quantified by ELISA (**e**) and correlated with tumor size, using simple linear regression statistics (**f**). Sera were further analyzed for the presence of antibodies against human GDF-15 (**e**). Sera were further tested for the presence of antibodies against

human GDF-15 (**g**). **h**. GDF-15 serum levels determined by ELISA (**e**) were correlated with the absence or presence of anti-human GDF-15 antibodies. **i.** C57Bl/6NCrl-mice were subcutaneously injected with MC38^{blank} or MC38$^{tghGDF-15}$ cells. The resulting tumors were allowed to grow in the right flank up to a volume of 1,000 mm^3. Tumor-infiltrating immune cells were assessed by immunocytochemistry. Shown are pictures for CD45$^+$ and CD8$^+$ TILs in mice euthanized on day 28 after tumor inoculation. **j,k.** Based on immunohistochemical staining, percentages of CD45$^+$ (**j**) and CD8$^+$ (**k**) cells were determined in MC38^{blank} or MC38$^{tghGDF-15}$ tumors that had grown to a volume of 1,000 mm^3. Due to the more frequent rejection of MC38^{blank} tumors, and to animals being lost for non-tumor-related reasons, only 3 tumors could be evaluated in the MC38^{blank} group, whereas 6 MC38$^{tghGDF-15}$ tumors were available for histological analysis. **l-o.** C57Bl/6NCrl-mice were subcutaneously inoculated with MC38^{blank} or MC38$^{tghGDF-15}$ colon cancer cells. One group of mice bearing MC38$^{tghGDF-15}$ tumors received an anti-human GDF-15 antibody twice weekly (20 mg/kg) while the other mice were mock-treated. Tumor growth curves based on 10 mice/group are shown in **l**. Dotted lines indicate the tumor size defined as termination criterion. **m-o**. Additional animals included in the study were euthanized when the tumors had reached a volume of 300 - 500 mm^3 (around day 23). Tumors were dissociated using the GentleMACS "Tumor Dissociation Kit" (Miltenyi) and CD45$^+$ (**m**), CD4$^+$ (**n**) and CD8$^+$ (**o**) cells were determined by flow cytometry. Again, rejection of MC38^{blank} tumors and loss of animals due to tumor-unrelated reasons resulted in an imbalance between the MC38^{blank} (n=2), the MC38$^{tghGDF-15}$ (n=3) and the MC38$^{tghGDF-15}$ groups treated with anti-GDF-15 antibody (n=5). **p-r.** C57Bl/6NCrl-mice were subcutaneously inoculated with MC38$^{tghGDF-15}$ tumors and treated from day 3 onwards twice per week with either vehicle, or anti-human GDF-15 antibody (20 mg/kg), or anti-PD-1 antibody (5 mg/kg), or anti-PD-1 and anti-human GDF-15 antibodies. Mice were euthanized on day 32 when at least 4 mice per group had tumors that allowed for immunohistochemical assessment. Shown are representative pictures (**p**) as well as a quantitation of tumor-infiltrating CD8$^+$ T cells (**q**) and an assessment of perinecrotic CD8$^+$ T cell infiltration (**r**). **s.** C57BL/6NCrl mice were subcutaneously inoculated with

5×10^{5} MC38^{blank} cells. Treatment with anti-PD-1 antibody (clone RMP1-14, Bio X Cell, 5 mg/kg) or vehicle control was initiated on day 3 and repeated on days 7, 10, 14, 17, 21, and 24. Tumor growth was monitored for 76 days. Individual growth curves and the corresponding Kaplan-Meier curves are shown**. t.** C57BL/6NCrl mice were subcutaneously inoculated with 5×10^{5} MC38$^{tghGDF-15}$ colon cancer cells and treated either with anti-PD-1 as in (**s**), anti-GDF-15 (20 mg/kg) on days 3, 7, 10, 14, 17, 21, 24, 27, 31 and 34, or a combination thereof. Survival was followed for 76 days. Individual tumor growth curves recorded over 76 days, and the corresponding Kaplan-Meier plots are shown for MC38$^{tghGDF-15}$ tumor-bearing C57BL/6NCrl mice treated with vehicle, anti-GDF-15, anti-PD-1 or anti-PD-1 and anti-GDF-15. Surviving mice showed no detectable tumor mass. **a,b,c,h,q** and **r** were analysed by two-sided unpaired t tests, **d** and **s** by log-rank (Mantel-Cox) test. For the experiment shown in **d**, a linear mixed-effects model providing a more detailed statistical analysis of tumor volume over time can be found in Supplementary Table 1. Wilcoxon-Mann-Whitney test was applied to **e,g,j** and **k**. Pearson's linear regression was calculated in **f**. In **m-o**, the very small number of tissues obtained from non-rejected tumors formed from MC38^{blank} cells has prompted us to refrain from statistical evaluation. In **t**, Cox proportional hazard models versus anti-PD-1 monotherapy (as reference treatment) were computed for all conditions. Hazard ratios, 95% confidence intervals and p values are shown. Individual tumor growth curves for **s** and **t** can be found in Supplementary Figure S4. Horizontal bars depict mean values ± SEM in **a,b,l**, and median values in **c,e,g,j,k,m,n,o,q,r**. All experiments were performed at least three times. In **a,b,d-t**, representative experiments are shown. Source data for Figures 3a-h,j-o,q-t are provided in the Source Data file. Flow cytometric gating strategies applied for Figures 3m-o are provided as Supplementary information.

Figure 4. GDF-15 blockade synergizes with anti-PD-1 in orthotopic Panc02 tumors and enhances T cell trafficking in syngeneic and humanized mice. In (**a,c,d,e**), 1x10^{4} luciferase-transgenic Panc02 cells were inoculated into the head of the pancreas of male albino C57Bl/6J mice. For surgery, mice were anesthetized with xylazin (20 mg/ml) and ursotamin (100 mg/ml) by i.p injection, shaved on the left lateral side and disinfected with braunol (aqueous povidone-iodine solution). Ten minutes after

injection of anesthetic, animals were checked for reflex between the toes to make sure about reaching the proper surgical tolerance. In case of positive reflex, an additional injection of 20 µl anesthetic intothe retroorbital venous plexus (i.v.) was performed. With a small lateral incision of the skin and peritoneum, abdominal cavity in the approximate position of the spleen was opened. Spleen and then the pancreas were externalized. Using a Hamilton syringe 30 µl of the Panc02 tumor cells were carefully injected into the pancreas. Successful injections were recognized by transparent bubble formation inside the pancreatic tissue. After suturing the peritoneum and skin with single stiches, they were kept in a warm environment and carefully monitored until they were awake from anesthesia. For pain relief metamizole (Novalgin) was injected subcutaneously and also added to drinking water. In (**b**), female albino C57Bl/6J mice were injected with $1x10^6$ Panc02-Luc cells into the tail of the pancreas. Following induction with isoflurane, a traverse incision was made over the spleen, followed by an incision in the underlying abdominal muscle. The spleen was gently removed from the abdominal cavity and secured along a second saline-wetted cotton bud to expose the underlying pancreas. The tail of the pancreas was located adjacent to the spleen. Using a 29G insulin syringe, 20 µL of a Matrigel-cell suspension was injected into the pancreas. The syringe was only removed from the pancreas for ~30-60 sec later after the Matrigel had solidified. The site of injection was inspected to ensure no leakage had occurred. The spleen and pancreas were carefully returned to the abdominal cavity. The abdomen and the skin were then be closed using a suture line followed by application of clips. Peri- and post-operative analgesia was administered; further post-operative analgesia was administered as deemed necessary. In either setting, animals were randomized by bioluminescence imaging on day 5, and treated twice-weekly with vehicle/anti-hGDF-15/anti-PD-1/ anti-hGDF-15+anti-PD1. Tumor load was assessed at least weekly via bioluminescent *in vivo* imaging. Tumor growth was assessed by relating the last available measurement to the signal during randomization. In (**a**), animals were euthanized on day 29 or, if needed, earlier, as indicated below the x-axis. Animals that failed to recover from the repeated anesthesia required for *in vivo* bioluminescence imaging were included based on the last available measurement. In (**b**), the

experiment was continued until day 35. Tumor growth was compared by Mann-Whitney test and corrected for multiple comparisons according to Bonferroni-Holm. The upper panels in a and b show the whole range of results, the lower panels provide a close-up on the range from -100% to +500% (**a**) or, respectively, -10% to +200% (**b**). (**c,d**). $CD4^+$ (**c**) and $CD8^+$ (**d)** T cell infiltration at day 12 were assessed by flow cytometry from disseminated tumor tissue (n = 6-8 mice/ group). To this aim, mouse tumor samples were dissociated using a gentleMACS™ dissociator with the corresponding Tumor Dissociation Kit. Tumor-infiltrating lymphocytes were stained by antibodies against CD45, CD3, CD4, and CD8. **e.** Using antibodies against CD8, Granzyme B and Foxp3, tissue sections were also stained on the automated IHC staining system Discovery XT and evaluated by an observer who was blinded with regard to the sample or treatment group. Representative stainings are shown. **f-i.** $2x10^5$ EMT6 murine breast cancer cells were orthotopically injected into the fourth mammary fat pad of female 7 to 10-week-old BALB/c mice. Mice were treated with anti-GDF-15 or isotype control on days 6, 9, and 12. On day 13, $CD3^+$ T cells were isolated from pooled spleens of BALB/c organ donor mice labeled with 5 µM carboxyfluorescein succinimidyl ester (CFSE). For adoptive transfer, $5x10^6$ labeled T cells were injected into the tail vein of EMT6 tumor-bearing mice. On day 14, tumors and tumor-draining lymph nodes were explanted. Infiltration of transferred $CD3^+$, $CD4^+$ and $CD8^+$ T cells into axillary (**f**) and brachial (**g**) lymph nodes was assessed by flow cytometry. Tumor size was measured on day 14, i.e. 24 h after adoptive T cell transfer, and the volume was calculated based on the ellipsoid formula: π/6 × (length × width2) by caliper measurement (**h**). Animals were then euthanized. Tumors and draining lymph nodes were harvested. Tumors were weighed after explantation (**i**). Presence and proliferation of CFSE-labeled $CD3^+$, $CD4^+$ and $CD8^+$ T cells in tumor- draining axillary (**f**) and brachial (**g**) lymph nodes was analyzed by flow cytometry. j-m. NOD/SCID/$\gamma c^{-/-}$ $FcR\gamma^{-/-}$ mice were reconstituted with human umbilical cord blood-derived $CD34^+$ hematopoietic stem cells and injected with the patient-derived HV-18-MK melanoma transplant that shows high endogenous GDF-15 expression. Mice then received either an anti-human GDF-15 (CTL-002) or an anti-FITC isotype control antibody (both used at 20 mg/kg, twice weekly). Animals were euthanized between day 24 and day 29, according to the termination criteria. GDF-15

serum levels were analyzed by ELISA and followed longitudinally across both studies (**j**). Day 24 serum levels were correlated with the size of those tumors that could be explanted without being disrupted (n=3). Pearson´s correlation coefficient and the corresponding p value are indicted (**k**). As anti GDF-15 antibody treatment affects clearance of GDF-15, only data from isotype control-treated mice are shown in **j** and **k**. Following tumor dissociation with collagenase D, infiltrating human $CD45^+$, $CD3^+$ and $CD19^+$ cells were determined by flow cytometry. Data from two independent experiments based on altogether 9 mice per treatment are shown in **l**. Different colors relate to different donors of $CD34^+$ HSC. In **m**, the composition of the $CD3^+$ T cell immune infiltrate on day 24 is shown in more detail for the four individual mice included in the second independent experiment. In **a,b**, tumor growth was analyzed by pairwise Mann-Whitney tests with Bonferroni-Holm correction. In **c,d,f,g,h,i**, groups were compared by unpaired Student´s t test. In **l** and **m**, Mann-Whitney-U test was performed, using overall cell percentages in **m**. Horizontal bars indicate mean±SEM in **c,d,f,g,h,i**, median values in **j,l,m.** Source data for Figures 4a-d,f-m are provided in the Source Data file. Flow cytometric gating strategies applied for Figures 4c,d,f,g,h,l and m are provided as Supplementary information.

Figure 5. GDF-15 expression correlated inversely with intratumoral T cell infiltration in brain metastases from melanoma patients and in HPV^+ oropharyngeal squamous cell carcinomas. a,b. Formalin-fixed paraffin-embedded (FFPE) tissues from 70 patients with melanoma brain metastases were collected and processed as tissue micro arrays (TMAs). All specimens were obtained from the cancer registry tumor bank “Blut-und Gewebebank zur Erforschung des malignen Melanoms” (Department of Dermato-Oncology, University Hospital Tübingen, Germany) and approved by the local ethics committee. Shown are exemplary tissue sections from melanoma brain metastases having no (upper panel) or high (lower panel) GDF-15 immunoreactivity, which were stained by immunohistochemistry for GDF-15 and for the T cell marker proteins CD3 and CD8 (**a**). **b-d.** All stained samples were scored according to the frequency of positive cells related to all cells (as percentage) on the stained TMA core. For hGDF-15 expression, a score was based on frequency (0-1% →score 0; 1-10% →score 1; 10-25% →score 2; 25-50% →score 3; >50% →score 4) multiplied

with staining intensity (weak→1, moderate→2, strong→3), resulting in an ordinal scaled hGDF-15. score (0, 1, 2, 3, 4, 6, 8, 9, 12). Spearman´s rank-correlation coefficient (ρ) and p values are indicated for correlations between the ordinal hGDF-15 score and the respective immune infiltrate. A dotted trend line was added for visualization. **e-h**. GDF-15 serum levels were assessed from patients with HPV^+ or HPV^- oropharyngeal squamous cell carcinomas. Tumor-infiltrating $CD8^+$ T cells per area were quantitated for 37 HPV^+ tumors and correlated with the corresponding GDF-15 serum levels (**e**). **f-h.** GDF-15 serum levels were divided into two groups with either GDF-15 < 1.0 ng/ml or GDF-15 ≥ 1.0 ng/ml. Kaplan-Meier plots for disease-specific survival were plotted for these two groups for patients with OPSCC irrespective of HPV status HPV^+ (n=86) (**f**), as well as for HPV^- (n=32) (**g**) and HPV^+ (n=54) (**h)** OPSCC. Spearman´s rank- correlation coefficients (ρ) and p values are indicated for **b-e**. Dotted trend lines were added for visualization. Kaplan-Meier curves were compared by log-rank (Mantel-Cox) test (**f-h**). Source data for Figures 5b-h are provided in the Source Data file.

Figure 6. In human melanoma patients GDF-15 serum levels predict response to and survival under therapy with anti-PD-1 antibodies. a. GDF-15 levels were analyzed in pre-treatment sera from 37 melanoma patients at the baseline of treatment with the anti-CTLA-4 antibody ipilimumab. GDF-15 serum levels were then correlated with the patients' responses classified according to the RECIST (response evaluation criteria in solid tumours) v1.1 response evaluation criteria. **b.** Responders to anti-CTLA-4 treatment (n=11) were classified based on whether they had a durable response (survived ≥ 1 year), or whether they relapsed and died within the first year after treatment. GDF-15 serum levels are indicated for these two groups. Results based on further follow-up are color-coded for patients with durable responses. **c,d.** GDF-15 levels were analyzed in pre-treatment sera from 29 melanoma patients at the baseline of treatment with the anti-PD-1 antibody pembrolizumab (Zurich cohort). GDF-15 serum levels were then correlated with the patients' responses classified according to the response evaluation criteria in solid tumors, version 1.1 (RECIST v1.1). In **c**, patients with ongoing responses at the time of analysis are demarked by black circles, all other patients are indicated by grey circles. In **d**, response to anti-PD-1 treatment (binary variable) was analyzed by logistic regression. For the response

to treatment as assessed by RECIST1.1 criteria after 4 months, a model was fitted with GDF-15 expression as continuous predictor. **e.** Overall survival of 34 patients was analyzed by Cox proportional hazards model with GDF-15 as continuous predictor. A Kaplan-Meier plot using a cut-off value of 2.0 ng/ml GDF-15 which corresponds to a median split (n=17 patients with [GDF-15] < 2.0 ng/ml, n=18 patients with [GDF-15] ≥ 2.0 ng/ml) is shown for visualization. Censoring is indicated by vertical lines. **f-j.** Pre-treatment GDF-15 levels were analyzed in sera from the Tübingen-based cohort of 88 patients scheduled for treatment with anti-PD-1 (pembrolizumab: n=48; nivolumab: n=40). All blood samples were taken within days prior to the treatment with the anti-PD1 antibody. **f.** In 87/88 patients, responses were classified according to RECIST1.1 criteria, differentiating between complete response (CR; n=9), partial response (PR; n=23), stable disease (SD; n=13) and progressive disease (PD; n=42). Patients still alive at the time point of the last analysis are indicated by black circles. GDF-15 levels were compared between the different groups using Kruskal- Wallis test. One patient who relapsed and died from melanoma within months despite being classified as complete responder was treated as on outlier in the CR group and thus disregarded for this analysis. **g,h.** The relationship between GDF-15 serum levels in ng/ml (continuous predictor) and binary response to anti-PD-1 treatment was analyzed by logistic regression models in 86 patients scheduled for treatment with anti-PD-1. The binary response was either defined as CR+PR (response) vs. SD+PD (**g**), or CR+PR+SD (disease control/clinical benefit) vs. PD (**h**). Logistic curves as estimated by the model are shown (lines) together with the data (circles). The dotted vertical line indicates the GDF-15 concentration where the probability of treatment response is 0.5. Two extreme values with [GDF-15] > 100 ng/ml are displayed at the upper end of the scale (accepting a slightly distorted representation to allow for a better representation of the other values). **i.** Cumulative survival probabilities according to Kaplan-Meier were calculated for patients with GDF-15 < 2.0 ng/ml (n=60) and patients with GDF-15 ≥ 2.0 ng/ml (n=28) and compared using two-sided log-rank tests **j**. Similarly, Kaplan-Meier curves were calculated for four patient groups defined by GDF-15 and LDH levels (n=88 patients in total). Censoring is indicated by vertical lines. Source data for Figures **6a-j** are provided in the Source Data file.

Supplementary information on gating strategies for flow cytometry

Whole blood adhesion assay (Figure 1a,b)

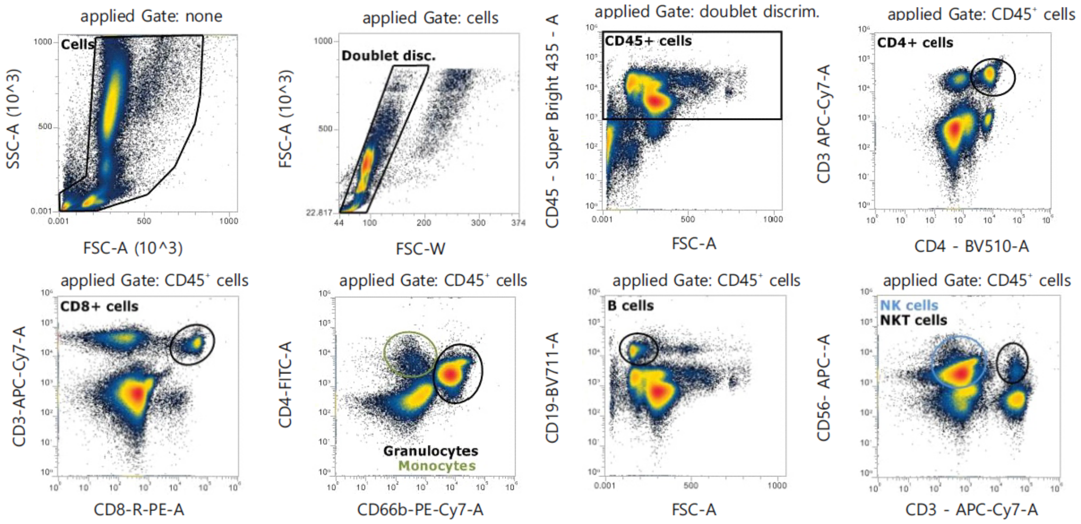

Ligand complex–based adhesion assay (LC-AA) from whole blood, Figure 2f,g, unstimulated cells

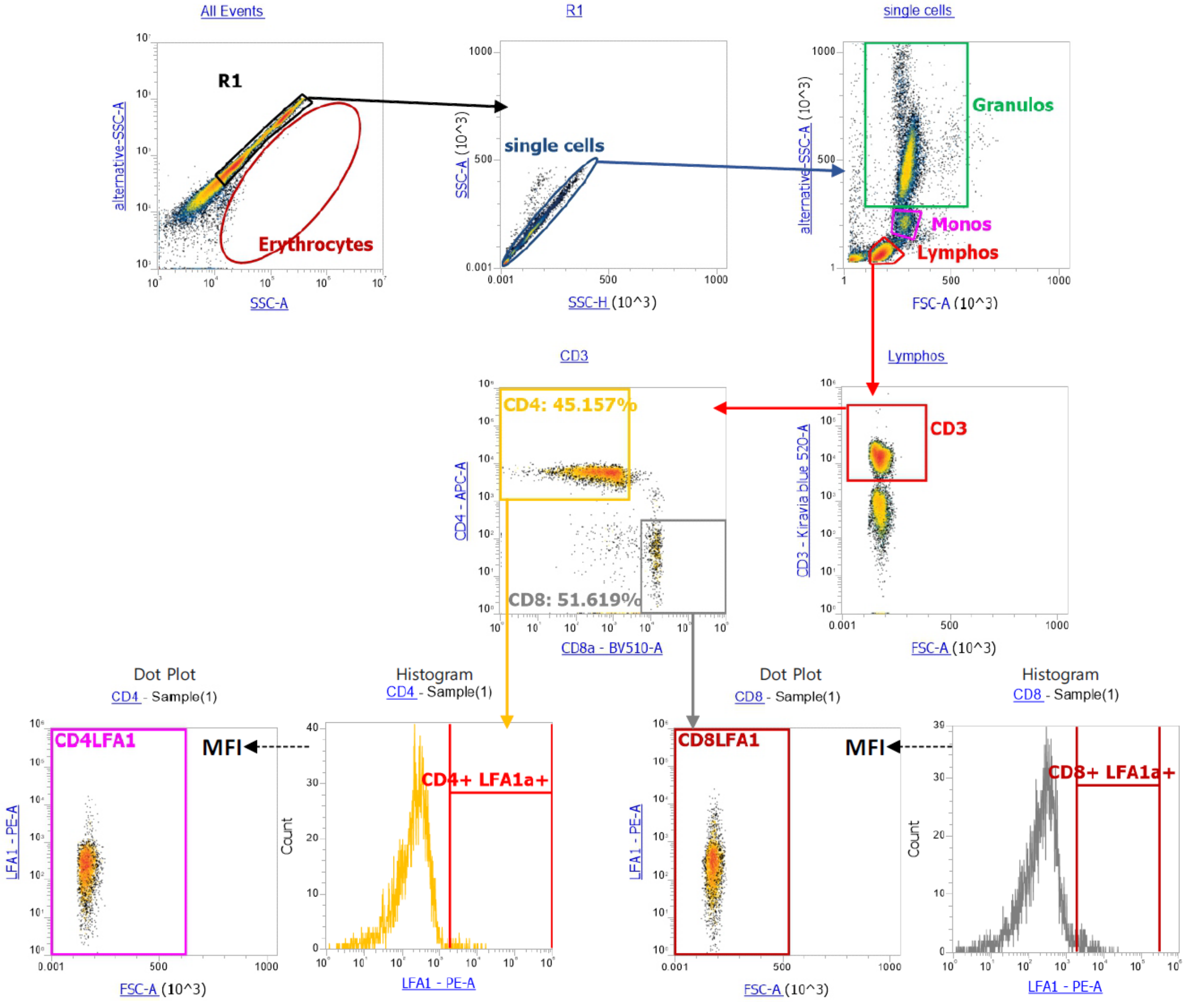

Ligand complex–based adhesion assay (LC-AA) from whole blood, Figure 2f,g, stimulated cells

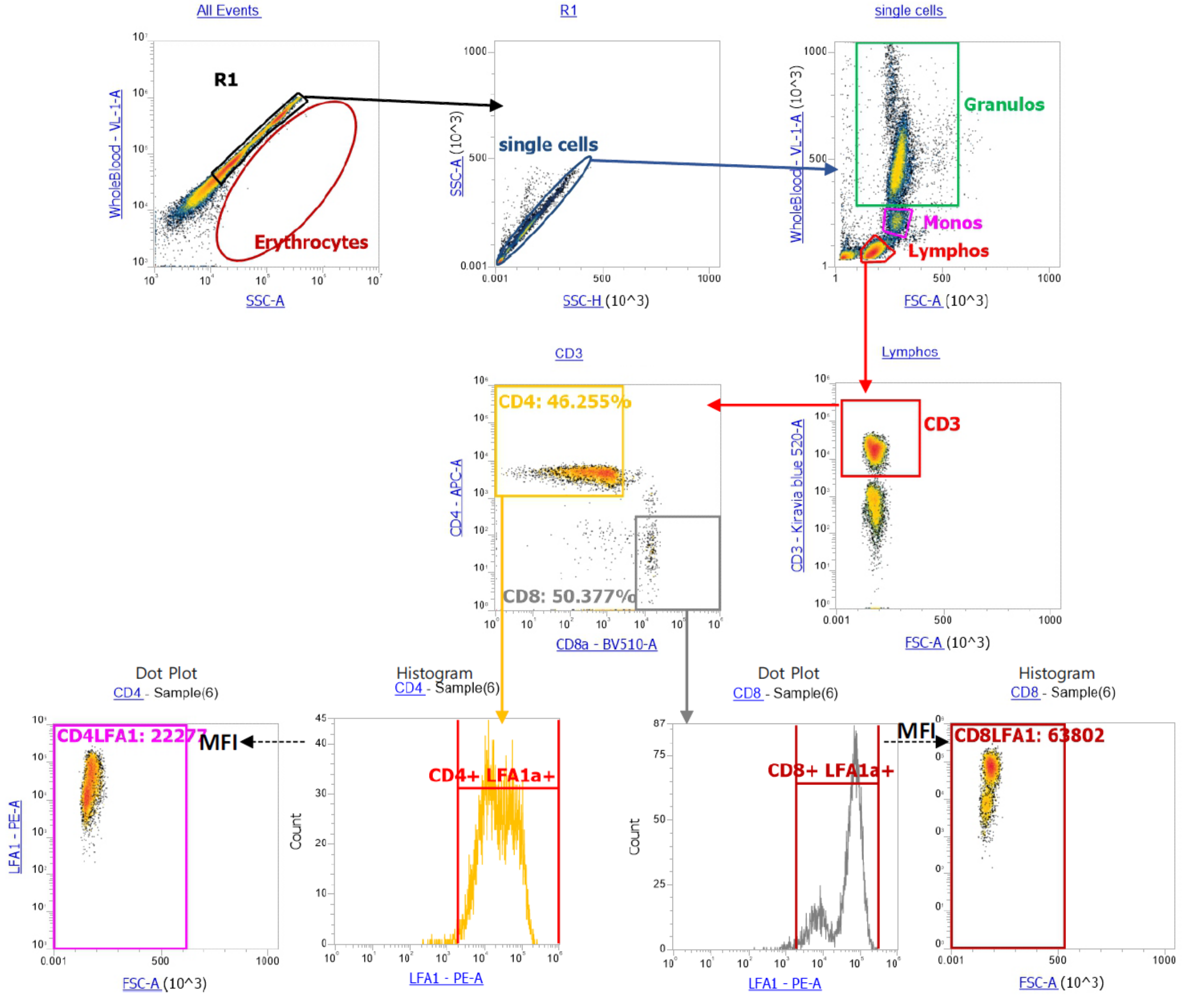

Immune infiltration in MC38$^{tghGDF-15}$ tumors (Figure 3m-o)

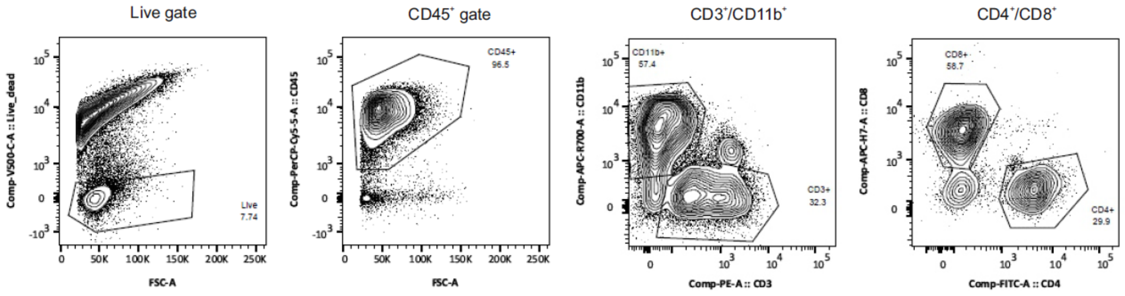

Immune infiltration in Panc02 tumors (Figure 4c-d, Supplementary Figure 5)

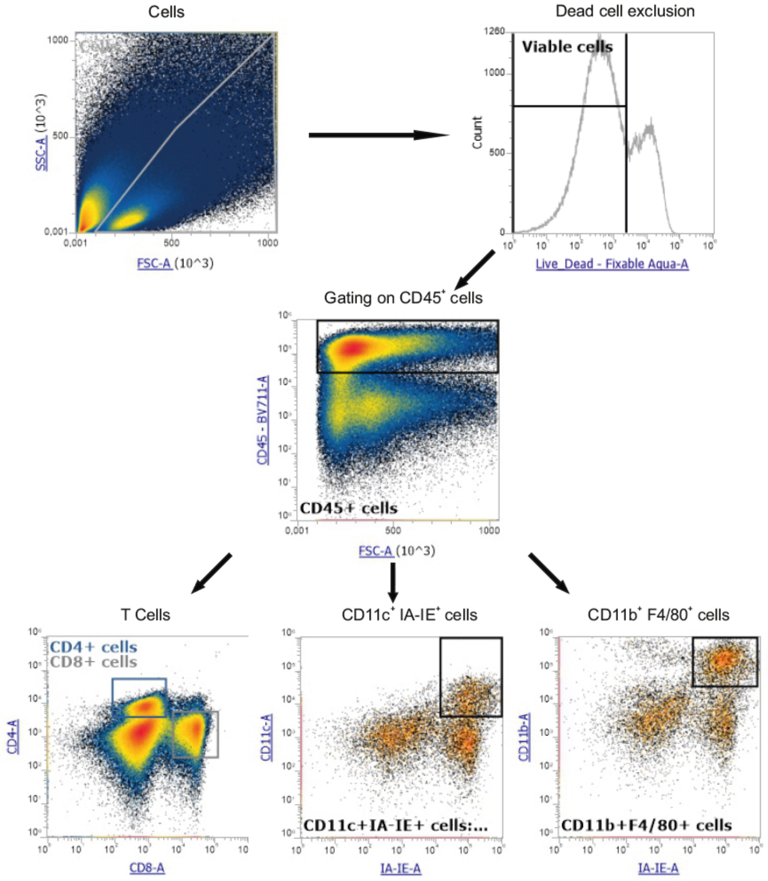

T cell infiltration in tumor-draining lymph nodes (Figure 4f,g,h)

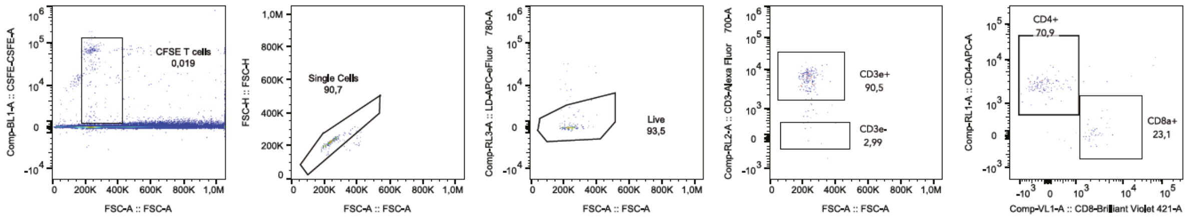

T cell infiltration in HV18MK xenograft tumors in humanized mice (Figure 4l,m)

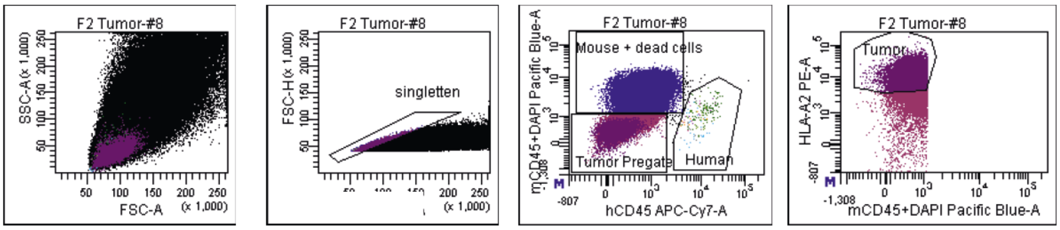

Supplementary information: Uncropped image of Western Blot shown in Figure 2k

Uncropped image of Western Blot (Figure 2k)

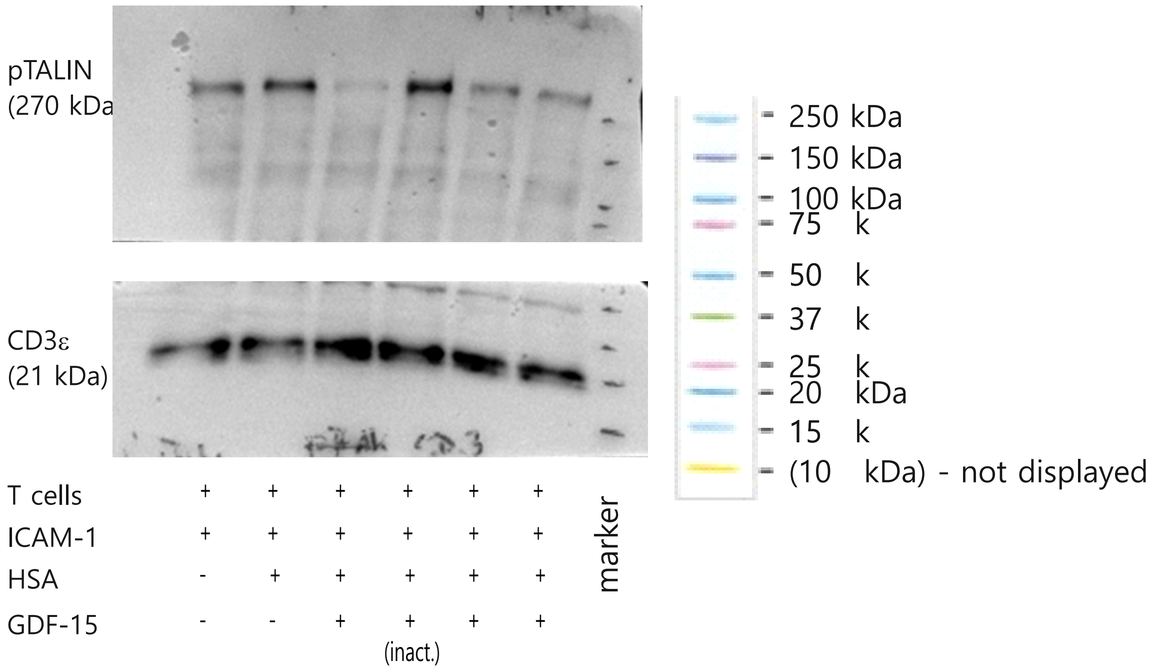

Supplement: Supplementary file 1 — Supplementary Information [file 41467_2023_39817_MOESM1_ESM.pdf]
