# Supplementary material for: Tumor-derived GDF-15 blocks LFA-1 dependent T cell recruitment and suppresses responses to anti-PD-1 treatment
Source: Nat Commun. 2023 Jul 20;14:4253. doi: 10.1038/s41467-023-39817-3 (PMC10359308; doi:10.1038/s41467-023-39817-3)
Supplement: Supplementary file 3 — Reporting Summary [file 41467_2023_39817_MOESM3_ESM.pdf]

nature portfolio

Corresponding author(s): Jörg Wischhusen

Last updated by author(s): May 17, 2023

Reporting Summary

Nature Portfolio wishes to improve the reproducibility of the work that we publish. This form provides structure for consistency and transparency in reporting. For further information on Nature Portfolio policies, see our Editorial Policies and the Editorial Policy Checklist.

Statistics

For all statistical analyses, confirm that the following items are present in the figure legend, table legend, main text, or Methods section.

n/a	Confirmed	
☐	☒	The exact sample size (*n*) for each experimental group/condition, given as a discrete number and unit of measurement
☐	☒	A statement on whether measurements were taken from distinct samples or whether the same sample was measured repeatedly
☐	☒	The statistical test(s) used AND whether they are one- or two-sided<br>*Only common tests should be described solely by name; describe more complex techniques in the Methods section.*
☐	☒	A description of all covariates tested
☐	☒	A description of any assumptions or corrections, such as tests of normality and adjustment for multiple comparisons
☐	☒	A full description of the statistical parameters including central tendency (e.g. means) or other basic estimates (e.g. regression coefficient) AND variation (e.g. standard deviation) or associated estimates of uncertainty (e.g. confidence intervals)
☐	☒	For null hypothesis testing, the test statistic (e.g. *F*, *t*, *r*) with confidence intervals, effect sizes, degrees of freedom and *P* value noted<br>*Give P values as exact values whenever suitable.*
☒	☐	For Bayesian analysis, information on the choice of priors and Markov chain Monte Carlo settings
☒	☐	For hierarchical and complex designs, identification of the appropriate level for tests and full reporting of outcomes
☐	☒	Estimates of effect sizes (e.g. Cohen's *d*, Pearson's *r*), indicating how they were calculated

Our web collection on statistics for biologists contains articles on many of the points above.

Software and code

Policy information about availability of computer code

Data collection	Only standard software (like the software provided by the manufacturer of the instrument used) and computer codes were employed for data collection. This included Attune Cytometric software and FlowJo for flow cytometry, Magellan for ELISA plate analysis, rapidSTORM 3.3 or dSTORM microscopy, iBright Analysis Software for Western Blot Analysis, EVOS® FL Cell Imaging System and Cell Profiler Software for flow adhesion assays, Living Image for in vivo bioluminescence analysis and DESeq2 package for pan-tumor analysis of gdf15 gene expression.
Data analysis	For data analysis, GraphPad Prism (version 9) and R (Versions 3.3.2 and 4.04) were used in addition to the specific software associated with the assay or measurement device like Attune Cytometric software and FlowJo (flow cytometry), Magellan (ELISA plate analysis), rapidSTORM 3.3 (dSTORM microscopy), iBright Analysis Software (Western Blot Analysis), EVOS® FL Cell Imaging System and Cell Profiler Software (flow adhesion assays), Living Image (in vivo bioluminescence analysis) and DESeq2 package (RNAseq database analysis).

For manuscripts utilizing custom algorithms or software that are central to the research but not yet described in published literature, software must be made available to editors and reviewers. We strongly encourage code deposition in a community repository (e.g. GitHub). See the Nature Portfolio guidelines for submitting code & software for further information.

Data

Policy information about availability of data

All manuscripts must include a data availability statement. This statement should provide the following information, where applicable:

- Accession codes, unique identifiers, or web links for publicly available datasets
- A description of any restrictions on data availability
- For clinical datasets or third party data, please ensure that the statement adheres to our policy

Source data for Figures 1a-j, 2a-g,j,l, 3a-h,j-o,q-t, 4a-d,f-m, 5b-h, 6a-j, S1a, S2a-i, S3c, S4a-f, S5a-c, S6a, S7a-f and S8a are provided as source data file. An unprocessed image of the Western Blot shown in Figure 2k and flow cytometric gating strategies applied for Figures 1a,b, 2f,g, 3m-o, 4c,d,f,g,h,l,m and S5a,b are provided as Supplementary information. Gene expression profiles from the Zurich cohort have been deposited under https://www.ncbi.nlm.nih.gov/geo/query/acc.cgi?acc=GSE198776. Additional questions will be answered by the corresponding author on reasonable request.

Human research participants

Policy information about studies involving human research participants and Sex and Gender in Research.

Reporting on sex and gender	Effects of combined GDF-15 blockade and anti-PD-1 treatment were shown in male and female mice with Panc02 tumors (Figures 4a,b). T cells used for mechanistic analyses came from anonymized male or female donors, with no information revealed to the investigators. After use, samples were discarded. Therefore, no further sex-specific analyses can be performed in this part of the manuscript.<br>Surplus sera obtained during routine blood draws at baseline of Ipilimumab treatment were available from 22 male and 15 female patients with stage IV melanoma at Würzburg University Hospital (age range: 36-79, median age: 68 years).<br>Surplus sera from 34 melanoma patients (25 male, 9 female, 26-80 year old, median age: 58 years) at baseline of pembrolizumab treatment were collected at the University Hospital Zurich (USZ) Biobank during routine blood draws from consenting metastatic melanoma patients. Surplus sera from 88 melanoma patients (54 male, 34 female, age range: 27-83 years, median age: 62 years) at the baseline of anti PD-1 treatment (pembrolizumab in 48, nivolumab in 40 patients) were collected in the Department of Dermatology at Tübingen University Hospital. All patients with histologically confirmed melanoma were identified in the Central Malignant Melanoma Registry (CMMR) database. Multi-variate analysis (Figure S7) revealed no statistically significant impact of sex on survival under therapy in this cohort. Likewise, a separate analysis of survival dichotomized by high/low GDF-15 serum levels showed no sex-specific differences (Supplementary Figure 7h).<br>Analyses relating to 86 patients (53 male, 33 female) with oropharyngeal squamous cell carcinoma were performed on samples from a larger study "Identification of immune response against HPV and p53 antigen in patients with a squamous cell tumor arising from the head and neck region" (P07-112). Due to the (compared to melanoma) lower number of patients with elevated GDF-15 results, the subgroup of female patients with elevated GDF-15 comprises just 5 samples, only one of them falling into the subgroup of female, HPV+ patients with GDF-15 > 1.0 ng/ml. Separate analyses for male and female patients from the complete cohort, the HPV- subgroups and the HPV+ GDF-15 low subset, revealed no sex-specific effects. Still, as this question has not been in the focus of our research, we prefer to avoid any poorly substantiated statements.
Population characteristics	See above. Inclusion criteria were a confirmed diagnosis, informed consent, availability of sera, and (in the case of melanoma patients) treatment with an immune checkpoint inhibitor. For the OPSCC cohort (86 patients, 53 male, 33 female), an aggregated and a separate analysis based on the respective subtype of the disease (54 of the HPVpos, 32 of the HPVneg subtype) Patient enrolment was from November 2007 until November 2015.<br>Surplus sera obtained during routine blood draws at baseline of ipilimumab treatment were available from 22 male and 15 female patients with stage IV melanoma at Würzburg University Hospital (age range: 36-79, median age: 68 years). Patients had received up to 4 applications of ipilimumab as monotherapy between 2011 and 2014 and were followed-up subsequently.<br>Surplus sera from 34 melanoma patients (25 male, 9 female, 26-80 year old, median age: 58 years) at baseline of pembrolizumab treatment were collected at the University Hospital Zurich (USZ) Biobank.<br>For the Tübingen melanoma cohort with sera from 88 melanoma patients (54 male, 34 female, age range: 27-83 years, median age: 62 years) at the baseline of anti PD-1 treatment (pembrolizumab in 48, nivolumab in 40 patients), a multivariate analysis was performed to assess effects of age, sex, line of treatment, further biomarkers (S100B and LDH) and presence or absence of brain metastasis.<br>However, as all these were retrospective analyses based on surplus serum samples, patients could not be characterized as this would have been done in a prospective, interventional clinical trial. Multi-variate analysis showed that age, sex, and line of treatment had no significant impact on outcome. Genotypic information e.g. on BRAF mutations were not available to the lead investigators of the present study.
Recruitment	All patient samples were stored surplus sera, which were retrospectively analyzed in a blinded fashion. No patients were specifically recruited for the present study. All available serum collections were analyzed in full and compared within the respective cohort. Thus, recruitment bias can be excluded.
Ethics oversight	86 Patients with histologically confirmed oropharyngeal squamous cell carcinoma were included at Leiden University Medical Center (LUMC) after they had signed the informed consent. The presented study was part of a larger observational study entitled: "Identification of immune response against HPV and p53 antigen in patients with a squamous cell tumor arising from the head and neck region" (P07-112). The study was approved by the Medical Ethics Committee Leiden The Hague Delft and was in agreement with the Dutch law. Patient enrolment was from November 2007 until November 2015.<br>Surplus sera obtained during routine blood draws at baseline of ipilimumab treatment were available from 22 male and 15 female patients with stage IV melanoma at Würzburg University Hospital. The retrospective analysis of GDF-15 levels was

approved by the Ethik-Kommission der Universität Würzburg (file number 20210310 01).
Surplus sera from 34 melanoma patients at baseline of pembrolizumab treatment were collected at the University Hospital Zurich (USZ) Biobank during routine blood draws from consenting metastatic melanoma patients according to institutional review board (Ethik-Kommission der Universität Zürich) approval (BASEC-Nr.PB_2017-00494) and following the Declaration of Helsinki on Human Rights. The University Research Priority Program in Translational Cancer Research (URPP) biobank processed the surplus material according to standard operating procedures established for routine biobanking at the USZ. Blood chemistry measurements were conducted by the routine hematology lab at the USZ according to standard procedures. Hemolysis prevented the determination of LDH levels in 4 samples.
Surplus sera from 88 melanoma patients at the baseline of anti PD-1 treatment were collected in the Department of Dermatology at Tübingen University Hospital. All patients with histologically confirmed melanoma were identified in the Central Malignant Melanoma Registry (CMMR) database 68. All patients had given written informed consent to have clinical data recorded by the CMMR registry. The Ethik-Kommission an der Medizinischen Fakultät der Eberhard-Karls-Universität und am Universitätsklinikum Tübingen has approved the study (ethical vote 125/2015BO2).

Note that full information on the approval of the study protocol must also be provided in the manuscript.

Field-specific reporting

Please select the one below that is the best fit for your research. If you are not sure, read the appropriate sections before making your selection.

☒ Life sciences ☐ Behavioural & social sciences ☐ Ecological, evolutionary & environmental sciences

For a reference copy of the document with all sections, see nature.com/documents/nr-reporting-summary-flat.pdf

Life sciences study design

All studies must disclose on these points even when the disclosure is negative.

Sample size

Sample size is indicated in the respective figure legends. In vitro experiments were repeated at least three times. Due to the part explorative and part retrospective character of the study, sample size estimates generally followed established laboratory standards with at least three repeats. In some mouse experiments (Figures 3j,k and 3m-o), the originally intended animal number of 6 mice/group to assess immune infiltration study were not attained due to spontaneous tumor rejections and loss of animals for tumor-unrelated reasons. However, as our committee for the conduct of research involving animals does not allow for including surplus mice to compensate for such losses, we had to accept that these experiments were underpowered. On the other hand, showing concordant data from different models still helps to develop a scientifically convincing picture, as the various findings support each other.
Clinical correlations were retrospectively analyzed from surplus samples. Consequently, the sample number in these cohorts was based on availability and an indirect consequence of the original study size cohort.

Data exclusions

Experimental data were included when the respective experiment met the quality criteria (positive and negative controls indicating that the experiment worked, reasonable standard errors). The only patient excluded in the clinical analyses was a melanoma patient (in Figure 6f) who had been staged with a complete response, but who nevertheless died from melanoma within weeks. As the assessment at staging was to be doubted, excluding this patient appeared to be the correct option. This is also indicated in the manuscript.

Replication

Key experiments have been replicated by independent investigators in different laboratories. Flow adhesion assays were performed by different investigators in 4 different laboratories. The Panc02 mouse model was performed in-house and at a CRO. Likewise, animal experiments with gene-modified MC38 cells were modified in-house and at a CRO. Results were concordant. To validate the important clinical findings, two independent cohorts of melanoma patients were analyzed in a blinded manner. Further experiments were repeated at least 3 times. Again, the consistency of the results obtained from different assays allows a cross-validation. Most importantly, the concept of blocking GDF-15 to improve immune infiltration in tumors and induce clinical responses has already been translated in a clinical phase 2 study. (Key data from the phase 1 trial will be disclosed in an oral presentation at ASCO 2023 in Chicago.) As the scientific concept is strongly supported by the clinical observations, the pre-clinical work has been validated in the best possible way.

Randomization

In animal experiments, tumors were allowed to grow for several days before animals were randomized into the different treatment groups. Human samples were only analyzed in retrospect. All cohorts were analyzed in full. Comparisons (e.g. survival or immune infiltration of patients with low vs. high GDF-15) were made within the respective cohort. Randomization was thus not applicable for this part of our study.

Blinding

Investigators were blinded for all tissue or serum analyses (Figures 3i,3p,4e,5a-h, 6a-j). Likewise, animal experiments in Figure 3a-t and 4j-m were performed without disclosing the identity of the respective antibodies to the scientist who performed the animal study. Unblinding was performed by an independent investigator, mostly at a different institution.

Reporting for specific materials, systems and methods

We require information from authors about some types of materials, experimental systems and methods used in many studies. Here, indicate whether each material, system or method listed is relevant to your study. If you are not sure if a list item applies to your research, read the appropriate section before selecting a response.

Materials & experimental systems

n/a	Involved in the study	
☐	☒	Antibodies
☐	☒	Eukaryotic cell lines
☒	☐	Palaeontology and archaeology
☐	☒	Animals and other organisms
☒	☐	Clinical data
☒	☐	Dual use research of concern

Methods

n/a	Involved in the study	
☒	☐	ChIP-seq
☐	☒	Flow cytometry
☒	☐	MRI-based neuroimaging

Antibodies

Antibodies used

Most antibodies used were standard antibodies against human or murine leukocyte antigens. These include anti-human CD45-SuperBright 436 clone 2D1 (eBioscience, San Diego, CA), anti-human CD3-APC/Cy7 clone HIT3a (BioLegend), anti-human CD4-BrilliantViolet711 clone A161A1 (BioLegend), anti-human CD19-BrilliantViolet711 clone HIB19 (BioLegend), anti-human CD56-APC clone 5.1H11 (BioLegend), anti-human CD66b-PECy7 clone G10P5 (BioLegend), anti-human CD8-PE clone UCHT-4 (ThermoFisher Scientific), anti-human CD14-FITC clone MEM-15 (ThermoFisher Scientific), AF647-conjugated anti-human IgG antibody (ThermoFisher Scientific, used at 5 µg/ml), anti-human LFA-1 antibody mAb24 (ThermoFisher Scientific, used at 5 µg/ml) anti-human CD3-AF488 (ThermoFisher Scientific, used at 2 µg/ml), anti-human CD8a-AF532 (ThermoFisher Scientific, used at 2 µg/ml), anti-mouse CD45-BV711 (clone 30-F11, BioLegend), anti-mouse CD3-PE-Cy7 (clone 145-2C11, BioLegend), anti-mouse CD8-BV421 (clone 53-6.7; Biolegend), anti-mouse CD4 PerCP Cyanine5.5 (clone RM4-5; Invitrogen), anti-mouse CD11b-APC (clone M1/70, BioLegend), anti-mouse IA-IE-FITC (clone M5/114.15.2, BioLegend), anti-mouse F4/80-BV421 (clone BM8, BioLegend), anti-Ly-6C-PE (clone HK1.4, BioLegend), anti-mouse Ly-6G-BV605 (clone 1A8; Biolegend), anti-mouse CD11c-PE-Cy7 (clone N418; Invitrogen), anti-mouse CD3 AF 700 (clone 500A2, Biolegend), anti-mouse CD4 APC (clone GK1.5, Biolegend), anti-human phospho-Talin Ser425 (clone D2P2M, Cell Signaling), anti-human CD3ε (Clone UCHT1, Biolegend), anti-mouse IgG HRP (#7076, Cell signaling technology), anti-human GDF-15 (HPA011191 Sigma/Atlas, 1:50 diluted), anti-human CD3 (clone A0452, DAKO, Glostrup, Denmark, dilution 1:500), anti-human CD8 (clone C8/144B, DAKO, Glostrup, Denmark, dilution 1:100), anti-human FOXP3 (clone 236A/E7; eBioscience, San Diego, U.S.A., dilution 1:100), anti-mouse CD8a (clone 4SM15, eBioscience, 1:100 dilution), anti-mouse Granzyme B (clone E5V2L, Cell Signaling Technologies, 1:100 dilution), anti-mouse FoxP3 (clone D6O8R, Cell Signaling Technologies, 1:100 dilution) anti-human LFA-1 antibody TS1/18 (ThermoFisher Scientific # MA1810 used at 20 µg/ml), anti-human VLA-4 blocking antibody (BioXcell, Lebanon, NH, USA, # BE0071, used 20µg/ml) anti-human α4β7 integrin antibody (R&D Systems #MAB10078, used at 5µg/ml), anti-mouse PD-1 antibody (clone Rmp1-14, BioXCell, used at 5 mg/kg), IgG1κ MOPC-21 (BioLegend), mIG2a B12 anti-HIV (Evitria, Schlieren, Switzerland) and anti-Fluorescein [4-4-20 (enhanced)] Human IgG4 S228P (Absolute antibodies). Unless otherwise indicated, standard antibodies were used as indicated by the manufacturer. (As most antibodies are sold as units of 25 or 100 tests without indicating the actual concentration, we cannot indicate concentrations, in particular as concentrations are optimized for the respective batch.) As these well-established clones yield characteristic staining patterns when applied to human PBMC, murine splenocytes, or control tissues like placenta (for GDF-15) or tonsils (for leukocyte markers), no further validation beyond suitable positive and negative controls was performed. The anti-human GDF-15 antibody used in this manuscript was generated in female C57BL/6J GDF-15-/--mice kindly provided by Dr. Jens Strelau (Heidelberg, Germany). For experiments in humanized mice, a humanized version of this antibody was generated by grafting the complementarity determining regions on a hinge-stabilized human IgG4 backbone, followed by recombinant expression (Evitria, Schlieren, Switzerland).
For experiments in fully immune-competent mice, a surrogate antibody (0297.mIgG1) was produced by Evitria. The cdr sequences of this antibody have been published in patent WO 2020/039321 A2. Binding was validated using recombinant murine GDF-15 as well as GDF-15 deficient and GDF-15 overexpressing cell lines. To block GFRAL, clone 3P10, described in Suriben, R. et al. Antibody-mediated inhibition of GDF15-GFRAL activity reverses cancer cachexia in mice. Nat Med 26, 1264-1270 (2020), and in US20170306031A1 was used.

Validation

Standard antibodies required no specific validation. They yield characteristic staining patterns when applied to human PBMC, murine splenocytes, or control tissues like placenta (for GDF-15) or tonsils (for leukocyte markers). Therefore, performing suitable positive and negative controls was deemed sufficient to control the performance. The anti-human GDF-15 antibody used in this manuscript was generated in female C57BL/6J GDF-15-/--mice kindly provided by Dr. Jens Strelau (Heidelberg, Germany). The anti-GDF-15 antibodies used for animal studies were validated for binding affinity by surface plasmon resonance at NMI (Reutlingen, Germany). In addition, binding was also confirmed by ELISA and by semi-native Western Blotting. Specificity was tested by tissue cross-reactivity studies. Anti-GDF-15 and anti-GFRAL antibodies were validated via binding to recombinant target proteins and via binding to transfected cell lines.

Eukaryotic cell lines

Policy information about cell lines and Sex and Gender in Research

Cell line source(s)

huLEC were obtained from InSCREENeX, Braunschweig, Germany, HUVECs from Lonza, Basel, Switzerland or freshly isolated. MC38 cells were provided by Charles River laboratories. Panc02 and EMT6 cells were obtained from ATCC. The generation of firefly-luciferase expressing Panc02 cells has been described in Chopra M, Lang I, Salzmann S, Pachel C, Kraus S, et al. (2013) Tumor Necrosis Factor Induces Tumor Promoting and Anti-Tumoral Effects on PancreaticCancer via TNFR1. PLoS ONE 8(9): e75737. doi:10.1371/journal.pone.0075737. HEK293T cells were obtained from ATCC and subcultured in the lab.

Authentication

Cells used in our laboratory are authenticated via DNA fingerprinting by the Deutsche Sammlung von Mikroorganismen und Zellkulturen (DSMZ). Cells provided by Charles River laboratories are authenticated by various methods in-house by CRL (https://www.criver.com/products-services/biologics-testing-solutions/cell-line-characterization?region=3696).

Mycoplasma contamination	We routinely test all cell cultures in-house using the PlasmoTest™ from Invivogen, which is based on HEK-Blue™-2 cells mycoplasma sensor cells and the corresponding HEK-Blue™ Detection medium. No contaminations were detected.
Commonly misidentified lines (See ICLAC register)	No commonly misidentified cell lines were included in this study.

Animals and other research organisms

Policy information about studies involving animals; ARRIVE guidelines recommended for reporting animal research, and Sex and Gender in Research

Laboratory animals	For animal experiments, 7-week-old female C57BL/6NCrl (Charles River Laboratories #027) or NCIAth/nu mice (Charles River Laboratories #490), male albino C57Bl/6J mice (B6(C)Rj-Tyr c-c, Janvier Laboratories, Saint Berthevin, France, #SC-C57AL-M), 8-9 weeks old female C57BL/6 albino (C57BL/6BrdCrHsd-Tyrc) (Envigo, Venray, Netherlands, #103) or 7 to 10-week-old BALB/c inbred (BALB/cOlaHsd, Envigo, #16204F) mice were used. NOD/Scid/γc-/-/FcRγ-/- mice are bred in house in Erlangen. The founder mice of the colony were obtained from Jackson laboratories (#005557).Mice were housed in facilities where temperature was controlled between 22 and 25°C, humidity was maintained between 40 and 70%, and artificial lighting was maintained for 12 hours per day.
Wild animals	No wild animals were used in this study.
Reporting on sex	Sex is indicated in all mouse experiments. One study has been performed with male mice. For all other studies female mice were used. In the melanoma cohort with 88 patients at baseline of anti-PD-1 treatment, male patients (n=54) had a hazard ratio of 1.797 (95% CI: 0.910-3.547, p=0.091) over female (n=34) patients with regard to death from melanoma.
Field-collected samples	No field-collected samples were used in this study.
Ethics oversight	Animal experiments were approved by the governmental review board of the state of Bayern, Regierung von Unterfranken, under the authorization numbers 55.2.2-2532-410 and 55.2.2-2532-1242-12.

Note that full information on the approval of the study protocol must also be provided in the manuscript.

Flow Cytometry

Plots

Confirm that:

☒ The axis labels state the marker and fluorochrome used (e.g. CD4-FITC).

☒ The axis scales are clearly visible. Include numbers along axes only for bottom left plot of group (a 'group' is an analysis of identical markers).

☒ All plots are contour plots with outliers or pseudocolor plots.

☐ A numerical value for number of cells or percentage (with statistics) is provided.

Methodology

Sample preparation	Standard procedures were followed.
Instrument	Attune NxT
Software	FlowJo software (Treestar, version 10.8.1) and Attune Cytometric Software
Cell population abundance	Flow cytometry was used to confirm that the purity of isolated cells was >90% prior to flow adhesion assays, to identify adherent cells (Fig. 1a,b), to assess ICAM-1-Fc and mAb24 binding to CD4+ and CD8+ T cells (Fig. 2f,g) and to assess T cell infiltration in tumors (Figures 3m-o, 4c,d,f,g,h,l,m). No rare cell subsets like antigen-specific T cells were investigated. In Figures 4f,g,h,l,m some samples showed target cell percentages <1%, which could easily be detected by measuring a sufficient overall number of cells. Moreover, as target cells could be identified by CFSE-staining or anti-human CD45, cells were easy to detect.
Gating strategy	Only standard assays were performed. Thus, there are no primary Flow Cytometry plots included in the manuscript. Therefore, the only primary Flow Cytometry data shown serve to exemplify the gating strategies. As these are exemplary plots where the actual percentage of cells will change with each sample, we have abstained from providing numerical information and statistics (which would only apply to the specific sample shown).

☒ Tick this box to confirm that a figure exemplifying the gating strategy is provided in the Supplementary Information.
